# Thyroid function and risk of all-cause and cardiovascular mortality: a prospective population-based cohort study

**DOI:** 10.1007/s12020-020-02397-z

**Published:** 2020-07-06

**Authors:** Dion Groothof, Jose L. Flores-Guerrero, Ilja M. Nolte, Hjalmar R. Bouma, Eke G. Gruppen, Arjola Bano, Adrian Post, Jenny E. Kootstra-Ros, Eelko Hak, Jens H. J. Bos, Martin H. de Borst, Reinold O. B. Gans, Thera P. Links, Robin P. F. Dullaart, Stephan J. L. Bakker

**Affiliations:** 1 Department of Internal Medicine, Division of Nephrology, University of Groningen, University Medical Center Groningen, Groningen, The Netherlands; 2 Department of Epidemiology, University of Groningen, University Medical Center Groningen, Groningen, The Netherlands; 3 Department of Internal Medicine, Division of Acute Medicine, University of Groningen, University Medical Center Groningen, Groningen, The Netherlands; 4 Department of Clinical Pharmacy and Pharmacology, University of Groningen, University Medical Center Groningen, Groningen, The Netherlands; 5 Department of Internal Medicine, Division of Endocrinology, University of Groningen, University Medical Center Groningen, Groningen, The Netherlands; 6 Institute of Social and Preventive Medicine, Bern University Hospital, University of Bern, Bern, Switzerland; 7 Department of Cardiology, Bern University Hospital, University of Bern, Bern, Switzerland; 8 Department of Laboratory Medicine, University of Groningen, University Medical Center Groningen, Groningen, The Netherlands; 9 Groningen Research Institute of Pharmacy, Unit of PharmacoTherapy, Epidemiology, and Economics, University of Groningen, Groningen, The Netherlands

**Keywords:** Thyroid function, Euthyroid, Mortality risk, Cohort study, General population, Biomarker

## Abstract

**Purpose:**

Although thyroid hormones are irrefutably implicated in cardiovascular physiology, the impact of within-reference range variations of thyroid function on cardiovascular disease (CVD) remains unclear. Elucidating this is important, since it could foster preventive treatment and reduce global CVD burden. We therefore investigated the impact of within-reference range variations of thyroid function on all-cause and cardiovascular mortality.

**Methods:**

We included community-dwelling individuals aged 28–75 years from a prospective cohort study, without known use of thyroid-affecting therapy and with thyrotropin within reference range. Associations of thyroid function with mortality were quantified using Cox models and adjusted for sociodemographic and cardiovascular risk factors.

**Results:**

Mean (SD) age of the 6,054 participants (52.0% male) was 53.3 (12.0) years. During 47,594 person-years of follow-up, we observed 380 deaths from all causes and 103 from CVDs. Although higher thyrotropin was not associated with all-cause mortality (adjusted HR 1.02, 95% CI 0.92–1.14), point estimates for cardiovascular mortality diverged toward increased risk in younger (<72 years) participants (1.31, 1.00–1.72) and decreased risk in elderly (≥72 years) (0.77, 0.56–1.06). Higher free thyroxine (FT_4_) was associated with all-cause mortality (1.18, 1.07–1.30) and with cardiovascular mortality only in elderly (1.61, 1.19–2.18), but not in younger participants (1.03, 0.78–1.34). Higher free triiodothyronine (FT_3_) was associated with all-cause mortality in females only (1.18, 1.02–1.35). FT_3_ was not associated with cardiovascular mortality (0.91, 0.70–1.18).

**Conclusions:**

Community-dwelling elderly individuals with high-normal thyroid function are at increased risk of all-cause and cardiovascular mortality, reinforcing the need of redefining the current reference ranges of thyroid function.

## Introduction

Abnormal thyroid function represents a significant public health burden, with an estimated prevalence approaching 70 per 1,000 individuals [1]. The potential health consequences are devastating if left untreated [2, 3]. Clinical (overt) thyroid dysfunction is defined as thyrotropin and free thyroxine (FT_4_) outside the reference ranges, whereas having abnormal thyrotropin with normal FT_4_ is referred to as subclinical thyroid dysfunction. To date, the 2.5th and 97.5th percentiles of the distribution of thyroid hormones, garnered from an apparently healthy population, serve as the mainstay in the establishment of the reference ranges [4]. Recent studies, however, have instigated the debate as to whether this approach has need of revision when risks for clinical outcomes are pondered as well [5–8].

Cardiovascular diseases (CVDs), currently the prime cause of death in developed countries, represent such an outcome. Although a large proportion of CVD events is explained by established risk factors, an estimated 25% still remains elusive [9]. As thyroid hormones are critically implicated in the cardiovascular system [10], special attention was drawn to thyroid hormones as potential biomarkers capable of explaining this remnant risk. Indeed, abnormal thyroid function, either clinical or subclinical, is a well-recognized risk factor of CVD [11–15]. Whether this risk extends into the established reference ranges remains to be determined [5–8, 14, 16, 17], and this information is important since it could pave the way for potential preventive treatment, and hence reduce the global disease burden inflicted by CVDs.

The aim of this study was therefore to investigate the impact of variations within reference ranges of thyroid function on all-cause and cardiovascular mortality in community-dwelling participants from the general population-based Prevention of REnal and Vascular ENd-stage Disease (PREVEND) Study.

## Methods

### Study design and participants

**Fig. 1:**
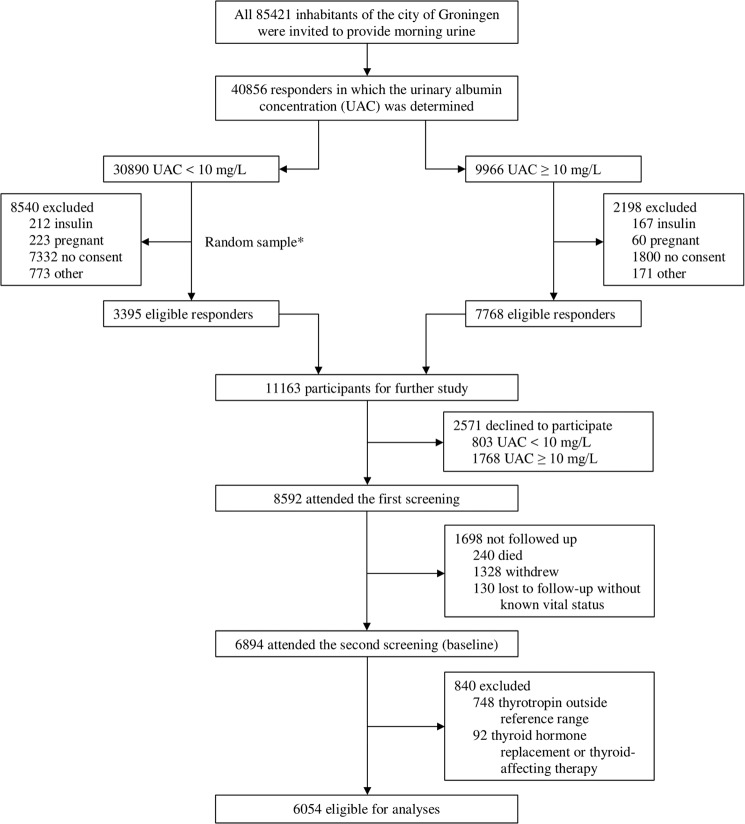
Flow of the participants through the study. *Size of the random sample was arbitrarily set at 3,395 (out of the 22,350 eligible participants) to obtain a total cohort size of ~10,000, taking into account a 15% nonparticipation rate

The PREVEND study investigates the risk factors for and the prevalence and consequences of microalbuminuria in otherwise healthy adults in the city of Groningen (The Netherlands). This cohort is specifically designed to monitor the long-term development of cardiac, renal, and peripheral vascular end-stage disease. The objectives and design have been described in detail elsewhere [18]. Briefly, all 85,421 inhabitants of the city of Groningen aged 28–75 years were invited, from 1997 to 1998, to participate in the study and were asked to complete a brief questionnaire and provide morning urine. The urinary albumin excretion (UAE) was determined in 40,856 responders. Pregnant women and participants with insulin-dependent diabetes mellitus were excluded. Participants with a UAE ≥ 10 mg/L (*n* = 7,768) were requested to participate in the cohort, of whom 6,000 were enrolled. In addition, a randomly chosen control group with a UAE of <10 mg/L (*n* = 3,395) was invited, of whom 2,592 were enrolled. These 8,592 participants constitute the PREVEND cohort. A second screening round took place from 2001 to 2003, encompassing 6,894 participants and was considered the “baseline” for the current study. Detailed information on the flow of participants through the study is provided in Fig. 1. We included 6,054 participants who fulfilled the inclusion criteria of not using thyroid hormone replacement or thyroid-affecting therapy (including levothyroxine, liothyronine, carbimazole, propylthiouracil, thiamazole, lithium salts, and amiodarone), having thyrotropin within the reference range of 0.27–4.20 mIU/L, and follow-up data on all-cause and cardiovascular mortality. The PREVEND study has been approved by the medical ethics committee of the University Medical Center Groningen and was undertaken in accordance with the Declaration of Helsinki. All participants provided written informed consent.

### Procedures

Each screening comprised two visits to an outpatient clinic separated by three weeks. Self-administered questionnaires concerning demographics, cardiovascular and renal disease history, smoking habits, and medication use were provided by all participants prior to the first visit. Information on medication use was combined with information from IADB.nl, a data base containing information of prescribed medication in public pharmacies in The Netherlands (http://www.iadb.nl/). Thyrotropin, thyroid function measurements, and antithyroid peroxidase were performed in baseline fasting serum samples stockpiled at −80 °C using the Roche Modular E170 Analyzer electrochemiluminescent immunoassays (Roche Diagnostics, Mannheim, Germany). Samples had been stored between 14 and 17 years and had not been thawed before. The expected values for thyrotropin (0.27–4.20 mIU/L), FT_4_ (12–22 pmol/L), and FT_3_ (3.1–6.8 pmol/L) as supplied by the manufacturer were set as the reference ranges. An antithyroid peroxidase titer of >34 kIU/L was considered positive.

### Outcomes

Study outcomes were incidence of all-cause mortality and cardiovascular mortality. Qualifying cardiovascular events comprised the International Classification of Diseases (ICD)-10 codes I10-I90. Data on mortality were obtained from municipality records and the cause of death was obtained from and classified by a physician from Statistics Netherlands and coded according to the 10th revision of the ICD.

### Statistical analyses

Survival time was calculated as the period between the second screening round (baseline) and events defined as death, loss to follow-up, or the end of the follow-up period (January 1, 2011), whichever came first. If a person had moved to an unknown destination, the date on which the person was dropped from the municipal registry was used as the census date. Linear trends across sex-stratified quartiles were determined using analysis of variance for normally distributed data, the Jonckheere–Terpstra test for skewed data, and the Mantel–Haenszel test of trend for categorical data. Crude linear associations between thyrotropin, FT_4_, and FT_3_ in all individuals and in younger (≤72 years) and elderly (>72 years) individuals separately were quantified by determining Pearson product–moment correlation coefficients. The rationale for this age cutoff is discussed below. We used Cox proportional hazards models to compute hazard ratios (HRs) of the association of thyrotropin, FT_4_, and FT_3_ with all-cause and cardiovascular mortality. Prior to entering the model, the correct covariate functional forms of thyrotropin, FT_4_, and FT_3_ were graphically determined by fitting a smoothing spline to the martingale residuals, obtained from a single Cox model fitting all covariates but thyrotropin, FT_4_, or FT_3_, along with a least squares multivariable regression line with thyrotropin, FT_4_, or FT_3_ as the dependent variable and all other covariates as independent variables [19]. The proportional hazards assumption was verified visually with plots of the scaled Schoenfeld residuals and formally by the Grambsch–Therneau goodness-of-fit test. All analyses were adjusted for the baseline values of all potential confounding variables (age, sex, current smoking, body-mass index, systolic blood pressure, use of antihypertensive and lipid-lowering medications, total cholesterol/high-density lipoprotein cholesterol ratio, triglycerides, type 2 diabetes, C-reactive protein, history of CVD, estimated glomerular filtration rate, and UAE) as fixed effects, which were selected on the basis of biological plausibility and previous literature. Point estimates are shown per SD increment in the predictor. Potential modification of the effect of thyrotropin or thyroid function on the risk of an event by age and sex was explored by including product terms in the model, which were allowed a permissive significance threshold of *α* = 0.10 [20]. Associations in the total population are shown along with sex-specific prospective associations. In addition, results of age-stratified associations are given to permit comparison with findings from previous studies, as they have primarily been conducted in elderly [5–7, 14, 16]. Cutoff values for the age strata were established through dichotomization of the cohort by the median age in participants who had succumbed to all-cause (70 years) and CVD (72 years), to obtain an equal number of events per stratum. The lower and higher age strata are referred to as “younger participants” and “elderly”, respectively. Only the results of the full models are reported (multivariable model). Multiple imputation using fully conditional specification was performed to obtain 20 imputed data sets with five iterations in the Gibbs sampler per data set [21], in which Rubin’s rules were applied to acquire pooled estimates of the regression coefficients and their standard errors across the imputed data sets [22]. All covariates had <5% missing values except for C-reactive protein (Supplementary Table 1). Statistical analyses were performed with R version 3.5.2 (Vienna, Austria) (http://cran.r-project.org/). A two-sided *P* value < 0.05 was judged significant.

### Sensitivity analyses

We conducted six distinct sensitivity analyses to evaluate the robustness of the findings. First, to account for potential bias caused by underlying autoimmune thyroid disease, we excluded 512 (8.3%) participants with a positive antithyroid peroxidase titer. Second, any potential bias caused by abnormal thyroid hormone tests was accounted for by excluding 221 (3.6%) participants with FT_4_ and/or FT_3_ concentrations outside the reference ranges. Third, to account for any potential bias caused by presence of cancer, we excluded 276 (4.5%) participants with prevalent malignancy since the first screening. Fourth, potential influence of pre-existing CVD on the associations was accounted for by excluding 389 (6.4%) participants with CVD at baseline. Fifth, to reduce the potential influence that preclinical frailty could have on the results (reverse causality), 51 (13.4%) deaths from all-causes and 20 (19.4%) from CVD that occurred during the first two years of follow-up were excluded. Last, to assess the impact of the cutoff values for the age strata in participants who had succumbed to all-cause (70 years) and CVD (72 years) on the obtained results, analyses were conducted with 65 years of age as the cutoff value for both the endpoint of all-cause mortality and cardiovascular mortality.

## Results

### Baseline characteristics

The mean (SD) age of the 6,054 participants was 53.3 (12.0) years of whom 3,149 (52.0%) were male. FT_4_ concentrations were substantially higher in males than females (*P* < 0.001), for which reason the baseline sociodemographic, clinical, and laboratory characteristics of the study participants are provided according to sex-stratified quartiles of FT_4_ (Table 1). Participants with higher FT_4_ concentrations were more likely to be younger or currently smoking, to have enjoyed higher education, to have a lower body-mass index, total cholesterol, total cholesterol/high-density lipoprotein cholesterol ratio, triglycerides, C-reactive protein, kidney function, and to be using antihypertensive drugs. Higher FT_4_ concentrations were not associated with a higher prevalence of CVD. Baseline concentrations of thyrotropin were negatively correlated with FT_4_ and FT_3_, while a positive correlation between FT_4_ and FT_3_ was observed; estimated correlation parameters *ρ* (95% CI) amounted to −0.16 (−0.18 to −0.13), –0.05 (−0.08 to −0.03), and 0.23 (0.20–0.25), respectively (Supplementary Table 2). In younger individuals, the correlation between FT4 and FT3 was particularly strong, with a value of 0.25 (0.22 to 0.27), while in elderly individuals the correlation between TSH and FT4 was particularly strong, with a value of −0.22 (−0.30 to −0.14), while the correlation between FT4 and FT3 was much weaker, with a value of 0.09 (0.00–0.17). After a median follow-up of 8.2 (interquartile range, 7.7–8.8) years, we observed 380 deaths from all causes, 103 of which were due to CVD.

**Table 1:** Baseline characteristics of the 6,054 participants according to sex-stratified quartiles of FT_4_

		Sex-stratified quartiles of FT_4_					
			I	II	III	IV	*P*_trend_
		Male	<14.5	14.5–15.8	15.9–17.2	>17.2	
		Female	<14.1	14.1–15.4	15.5–16.7	>16.7	
	All participants (*N* = 6,054)	Male	788	787	787	787	
FT4, pmol/L		Female	727	726	726	726	
Sociodemographic characteristics							
Age	53.3 (12.0)		54.0 (11.4)	53.5 (11.6)	52.7 (12.1)	52.9 (12.6)	0.002
Race							
Caucasian	5766 (95.2)		1452 (95.8)	1445 (95.5)	1439 (95.1)	1430 (94.5)	0.02
Negroid	60 (1.0)		18 (1.2)	18 (1.2)	13 (0.9)	11 (0.7)	
Asian	116 (1.9)		19 (1.3)	28 (1.9)	36 (2.4)	33 (2.2)	
Other	66 (1.1)		14 (0.9)	14 (0.9)	17 (1.1)	21 (1.4)	
Unknown	46 (0.8)		12 (0.8)	8 (0.5)	8 (0.5)	18 (1.2)	
Education							
Low	2612 (43.1)		693 (45.7)	677 (44.7)	638 (42.3)	604 (39.9)	0.001
Middle	1543 (25.5)		385 (25.4)	360 (23.8)	388 (25.6)	410 (27.1)	
High	1899 (31.4)		437 (28.8)	476 (31.5)	487 (32.2)	499 (33.0)	
Current smoking	1749 (28.9)		385 (25.4)	416 (27.5)	464 (30.7)	484 (32.0)	<0.001
Drinking alcohol, ≥10 g/day	1638 (27.1)		404 (26.7)	414 (27.4)	419 (27.7)	401 (26.5)	0.98
Type 2 diabetes	363 (6.0)		94 (6.2)	106 (7.0)	83 (5.5)	80 (5.3)	0.12
History of CVD	389 (6.4)		80 (5.3)	107 (7.1)	113 (7.5)	89 (5.9)	0.43
Thyroid function							
TSH, mIU/L	1.72 (0.82)		1.89 (0.87)	1.75 (0.83)	1.68 (0.78)	1.57 (0.75)	<0.001
FT3, pmol/L	4.8 (4.5–5.2)		4.7 (4.3–5.0)	4.8 (4.5–5.1)	4.9 (4.6–5.2)	5.0 (4.7–5.4)	<0.001
Anti-TPO, kIU/L	10.3 (8.1–13.8)		10.2 (8.0–13.9)	10.3 (7.9–13.5)	10.3 (8.1–13.7)	10.5 (8.3–14.1)	0.09
Body composition							
BMI, kg/m^2^	26.6 (4.3)		27.3 (4.5)	26.7 (4.3)	26.5 (4.3)	26.1 (4.1)	<0.001
Haemodynamics							
SBP, mmHg	126.2 (18.7)		126.2 (18.4)	125.9 (18.3)	126.1 (18.9)	126.5 (19.2)	0.62
DBP, mmHg	73.5 (9.2)		73.6 (9.4)	73.7 (9.1)	73.4 (9.1)	73.2 (9.1)	0.22
Lipid spectrum							
Total cholesterol, mmol/L	5.4 (1.0)		5.5 (1.1)	5.4 (1.0)	5.4 (1.0)	5.4 (1.1)	<0.001
HDL cholesterol, mmol/L	1.25 (0.31)		1.24 (0.32)	1.25 (0.31)	1.25 (0.31)	1.26 (0.30)	0.09
Total cholesterol/HDL	4.6 (1.3)		4.7 (1.4)	4.6 (1.3)	4.5 (1.3)	4.5 (1.3)	<0.001
Triglycerides, mmol/L	1.12 (0.81–1.61)		1.19 (0.83–1.71)	1.13 (0.81–1.67)	1.10 (0.80–1.56)	1.06 (0.79–1.50)	<0.001
Inflammation							
Hs-CRP, mg/L	1.36 (0.62–3.11)		1.46 (0.67–3.25)	1.30 (0.61–3.08)	1.41 (0.63–3.14)	1.26 (0.56–3.00)	0.01
Kidney function parameters							
eGFR_SCr-CysC_, mL/min/1.73 m^2^	90.8 (20.7)		91.2 (20.7)	91.3 (20.7)	91.2 (21.0)	89.5 (20.6)	0.02
UAE, mg/day	8.7 (6.1–15.8)		8.9 (6.1–17.2)	8.7 (6.0–15.6)	8.8 (6.1–15.3)	8.3 (6.0–15.6)	0.05
UAE categories							
<15 mg/day	4436 (73.3)		1080 (71.3)	1114 (73.6)	1126 (74.4)	1116 (73.8)	0.10
15–29.9 mg/day	818 (13.5)		206 (13.6)	217 (14.3)	201 (13.3)	194 (12.8)	
30–300 mg/day	707 (11.7)		199 (13.1)	166 (11.0)	160 (10.6)	182 (12.0)	
>300 mg/day	93 (1.5)		30 (2.0)	16 (1.1)	26 (1.7)	21 (1.4)	
Medication							
Antihypertensive drugs	1264 (20.9)		291 (19.2)	297 (19.6)	325 (21.5)	351 (23.2)	0.003
Lipid-lowering drugs	560 (9.3)		146 (9.6)	148 (9.8)	140 (9.3)	126 (8.3)	0.18

Data are mean (SD), median (IQR), or *n* (%)

*BMI* body-mass index, *CVD* cardiovascular disease, *CysC* serum cystatin C, *DBP* diastolic blood pressure, *eGFR* estimated glomerular filtration rate, *FT*_*3*_ triiodothyronine, *FT*_*4*_ free thyroxine, *HDL* high-density lipoprotein, *Hs-CRP* high-sensitive C-reactive protein, *SBP* systolic blood pressure, *SCr* serum creatinine, *TPO* thyroid peroxidase, *TSH* thyrotropin, *UAE* urinary albumin concentration

### Association of thyrotropin and thyroid function with all-cause mortality

Of the 380 deaths, 288 (75.8%) occurred in males and 92 (24.2%) in females. The association of thyrotropin with all-cause mortality was not statistically significant (HR 1.02, 95% CI 0.92–1.14; *P* = 0.65) and was neither modified by sex (Table 2) nor age (Table 3). Higher FT_4_ was associated with increased risk of all-cause mortality (1.18, 1.07–1.30; *P* = 0.001). This association was more pronounced in females (1.47, 1.19–1.82; *P* < 0.001) than in males (1.14, 1.01–1.27; *P* = 0.03) (*P*_interaction_ = 0.01). Moreover, higher FT_4_ was strongly associated with increased risk of all-cause mortality in elderly (≥70 years) (1.28, 1.11–1.48; *P* = 0.001) (Table 3). Although FT_3_ was not associated with all-cause mortality in the total population (Table 2), a strong effect modification by sex was observed (*P*_interaction_ = 0.006). Higher FT_3_ was associated with an increased risk of all-cause mortality in females (1.19, 1.06–1.34; *P* = 0.004), but not in males (0.92, 0.79–1.07; *P* = 0.26). This association was not modified by age (Table 3).

**Table 2:** Total and sex-stratified prospective associations of thyrotropin and thyroid function with all-cause and cardiovascular mortality

	TSH			FT_4_			FT_3_		
	HR (95% CI)	*P* value	*P*_interaction_^a^	HR (95% CI)	*P* value	*P*_interaction_^a^	HR (95% CI)	*P* value	*P*_interaction_^a^
All-cause mortality									
Total population *n*_events_/*n*_total_ = 380/6054									
Crude	0.93 (0.84–1.03)	0.18		1.29 (1.17–1.42)	<0.001		0.88 (0.78–1.00)	0.05	
Age- and sex-adjusted	0.99 (0.90–1.10)	0.89	0.90	1.24 (1.13–1.37)	<0.001	0.03	0.99 (0.89–1.09)	0.81	0.002
Multivariable model^b^	1.02 (0.92–1.14)	0.65	0.87	1.18 (1.07–1.30)	0.001	0.01	0.99 (0.89–1.10)	0.81	0.006
Men *n*_events_/*n*_total_ = 288/3149									
Crude	0.97 (0.85–1.09)	0.58		1.10 (0.99–1.24)	0.09		0.60 (0.51–0.70)	<0.001	
Age-adjusted	1.00 (0.89–1.13)	0.99		1.17 (1.04–1.31)	0.007		0.91 (0.78–1.07)	0.25	
Multivariable model^c^	1.00 (0.89–1.14)	0.95		1.14 (1.01–1.27)	0.03		0.92 (0.79–1.07)	0.27	
Women *n*_events_/*n*_total_ = 92/2905									
Crude	1.00 (0.82–1.22)	0.99		1.68 (1.37–2.05)	<0.001		1.11 (0.99–1.24)	0.08	
Age-adjusted	0.99 (0.82–1.19)	0.89		1.56 (1.28–1.90)	<0.001		1.18 (1.05–1.32)	0.005	
Multivariable model^c^	1.07 (0.89–1.30)	0.47		1.47 (1.19–1.82)	<0.001		1.18 (1.02–1.35)	0.02	
Cardiovascular mortality									
Total population *n*_events_/*n*_total_ = 103/6054									
Crude	0.89 (0.73–1.09)	0.26		1.40 (1.16–1.69)	<0.001		0.73 (0.57–0.93)	0.01	
Age- and sex-adjusted	0.97 (0.79–1.18)	0.75	0.87	1.34 (1.11–1.61)	0.002	0.40	0.86 (0.66–1.12)	0.27	0.46
Multivariable model^b^	0.98 (0.80–1.20)	0.86	0.84	1.23 (1.02–1.49)	0.03	0.22	0.91 (0.70–1.18)	0.48	0.67
Men *n*_events_/*n*_total_ = 83/3149									
Crude	0.92 (0.72–1.16)	0.48		1.21 (0.98–1.49)	0.07		0.50 (0.37–0.67)	<0.001	
Age-adjusted	0.96 (0.77–1.21)	0.73		1.29 (1.04–1.59)	0.02		0.83 (0.61–1.13)	0.27	
Multivariable model^c^	0.93 (0.73–1.18)	0.56		1.23 (1.00–1.52)	0.05		0.87 (0.64–1.18)	0.38	
Women *n*_events_/*n*_total_ = 20/2905									
Crude	1.02 (0.67–1.55)	0.93		1.82 (1.19–2.78)	0.005		0.92 (0.53–1.61)	0.78	
Age-adjusted	1.00 (0.67–1.50)	0.99		1.61 (1.07–2.42)	0.02		1.02 (0.59–1.76)	0.95	
Multivariable model^c^	1.17 (0.77–1.78)	0.47		1.46 (0.92–2.31)	0.10		0.96 (0.54–1.70)	0.89	

Point estimates are expressed per SD increment in the predictor

*CI* confidence interval, *FT*_*3*_ free triiodothyronine, *FT*_*4*_ free thyroxine, *HR* hazard ratio, *TSH* thyrotropin

^a^Evidence against the null hypothesis of no effect modification by sex on the association of the thyroid function parameter and the specific outcome concerned

^b^Adjusted for age, sex, current smoking, body-mass index, systolic blood pressure, use of antihypertensive drugs, total cholesterol/high-density lipoprotein ratio, ln triglycerides, use of lipid-lowering drugs, type 2 diabetes, high-sensitive C-reactive protein, history of cardiovascular disease, estimated glomerular filtration rate, and ln urinary albumin excretion

^c^Adjusted for all potential confounders except for sex

**Table 3:** Total and age-stratified prospective associations of thyrotropin and thyroid function with all-cause and cardiovascular mortality

	TSH			FT_4_			FT_3_		
	HR (95% CI)	*P* value	*P*_interaction_^a^	HR (95% CI)	*P* value	*P*_interaction_^a^	HR (95% CI)	*P* value	*P*_interaction_^a^
All-cause mortality									
Total population *n*_events_/*n*_total_ = 380/6054									
Crude	0.93 (0.84–1.03)	0.18		1.29 (1.17–1.42)	<0.001		0.88 (0.78–1.00)	0.05	
Age- and sex-adjusted	0.99 (0.90–1.10)	0.89	0.31	1.24 (1.13–1.37)	<0.001	0.21	0.99 (0.89–1.09)	0.81	0.81
Multivariable model^b^	1.02 (0.92–1.14)	0.65	0.55	1.18 (1.07–1.30)	0.001	0.46	0.99 (0.89–1.10)	0.81	0.85
Low age (<70 years)^c^ *n*_events_/*n*_total_ = 190/5304									
Crude	0.86 (0.74–1.00)	0.05		1.16 (1.00–1.34)	0.05		1.00 (0.87–1.15)	0.98	
Age- and sex-adjusted	0.89 (0.77–1.03)	0.13		1.15 (1.00–1.32)	0.05		0.99 (0.87–1.12)	0.82	
Multivariable model^b^	0.99 (0.85–1.15)	0.86		1.11 (0.97–1.28)	0.14		0.98 (0.86–1.12)	0.79	
High age (≥70 years)^c^ *n*_events_/n_total_ = 190/750									
Crude	1.04 (0.91–1.19)	0.57		1.35 (1.18–1.55)	<0.001		1.06 (0.88–1.28)	0.55	
Age- and sex-adjusted	1.10 (0.96–1.26)	0.18		1.36 (1.18–1.57)	<0.001		1.02 (0.84–1.24)	0.85	
Multivariable model^b^	1.07 (0.93–1.24)	0.32		1.28 (1.11–1.48)	0.001		1.04 (0.86–1.25)	0.70	
Cardiovascular mortality									
Total population *n*_events_/*n*_total_ = 103/6054									
Crude	0.89 (0.73–1.09)	0.26		1.40 (1.16–1.69)	0.001		0.73 (0.57–0.93)	0.01	
Age- and sex-adjusted	0.97 (0.79–1.18)	0.75	0.09	1.34 (1.11–1.61)	0.002	0.03	0.86 (0.66–1.12)	0.27	0.16
Multivariable model^b^	0.98 (0.80–1.20)	0.86	0.04	1.23 (1.02–1.49)	0.03	0.06	0.91 (0.70–1.18)	0.48	0.28
Low age (<72 years)^c^ *n*_events_/*n*_total_ = 51/5551									
Crude	1.07 (0.82–1.40)	0.62		1.16 (0.88–1.53)	0.28		0.98 (0.73–1.32)	0.90	
Age- and sex-adjusted	1.11 (0.86–1.44)	0.42		1.13 (0.87–1.47)	0.35		0.98 (0.75–1.27)	0.87	
Multivariable model^b^	1.31 (1.00–1.72)	0.05		1.03 (0.78–1.34)	0.85		1.00 (0.77–1.30)	0.98	
High age (≥72 years)^c^ *n*_events_/*n*_total_ = 52/503									
Crude	0.80 (0.59–1.07)	0.13		1.55 (1.19–2.03)	0.001		0.77 (0.52–1.15)	0.20	
Age- and sex-adjusted	0.82 (0.60–1.11)	0.20		1.63 (1.23–2.17)	0.001		0.72 (0.48–1.09)	0.12	
Multivariable model^b^	0.77 (0.56–1.06)	0.11		1.61 (1.19–2.18)	0.002		0.81 (0.54–1.21)	0.30	

Point estimates are expressed per SD increment in the predictor

*CI* confidence interval, *FT*_*3*_ free triiodothyronine, *FT*_*4*_ free thyroxine, *HR* hazard ratio, *TSH* thyrotropin

^a^Evidence against the null hypothesis of no effect modification by age on the association of the thyroid function parameter and the specific outcome concerned

^b^Adjusted for age, sex, current smoking, body-mass index, systolic blood pressure, use of antihypertensive drugs, total cholesterol/high-density lipoprotein ratio, ln triglycerides, use of lipid-lowering drugs, type 2 diabetes, high-sensitive C-reactive protein, history of cardiovascular disease, estimated glomerular filtration rate, and ln urinary albumin excretion

^c^Cutoff values for the age strata were established through dichotomization of the cohort by the median age in participants who had succumbed, to obtain an equal amount of events per stratum

All sensitivity analyses yielded similar results for associations of thyrotropin and FT_4_ with mortality (Supplementary Tables 3–13). Exclusion of participants with a positive antithyroid peroxidase titer, circulating FT4 and/or FT3 outside the reference ranges, or with prevalent malignancy since the first screening obliterated the association of higher FT_3_ with all-cause mortality in females (Supplementary Tables 3, 5, and 7). Other sensitivity analyses on the associations of thyrotropin and thyroid function parameters with mortality provided similar results (Supplementary Tables 3–13).

### Association of thyrotropin and thyroid function parameters with cardiovascular mortality

**Fig. 2:**
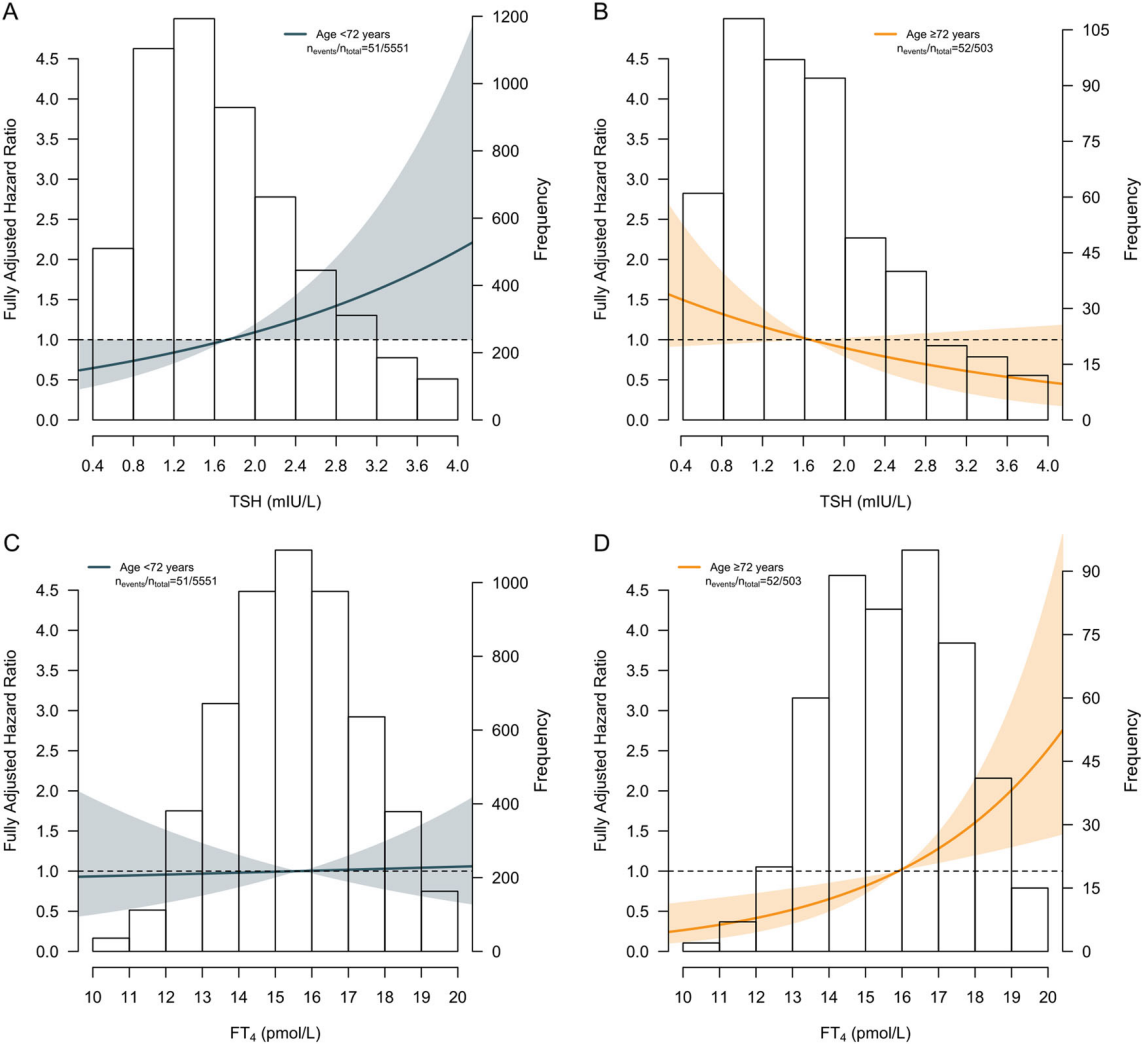
Graphical representation of the survival estimates of thyrotropin and thyroxine for cardiovascular mortality stratified by age. **a** shows the impact of thyrotropin on the risk of cardiovascular mortality in participants younger than 72 years and **b** in participants aged 72 years or older. **c** shows the impact of FT_4_ on the risk of cardiovascular mortality in participants younger than 72 years and **d** in participants aged 72 years or older. The lines represent the risk of cardiovascular mortality. The areas about the lines represent the 95% confidence interval associated with the line. The median thyrotropin and FT_4_ concentration was chosen as a reference in the upper two and lower two panels, respectively. The distribution of thyrotropin and FT_4_ in both age strata is depicted by the histogram in the background. Cutoff values for the age strata were established through dichotomization of the cohort by the median age in participants who had succumbed to cardiovascular disease, to obtain an equal amount of events per stratum. FT_4_ thyroxine, TSH thyrotropin

Of the 103 deaths due to CVD, 83 (80.6%) occurred in males and 20 (19.4%) in females. No association was found between thyrotropin and cardiovascular mortality in the total population (HR 0.98, 95% CI 0.80–1.20; *P* = 0.86) and this association was not modified by sex (Table 2). Interestingly, opposite effects were observed between age strata (*P*_interaction_ = 0.04). Higher thyrotropin showed a trend toward increased risk of cardiovascular mortality in younger (<72 years) participants (1.31, 1.00–1.72; *P* = 0.05), whereas an opposite trend was observed in elderly (≥72 years) (0.77, 0.56–1.06; *P* = 0.11) (Table 3 and Fig. 2a, b). Higher FT_4_ was associated with increased risk of cardiovascular mortality in the total population (1.23, 1.02–1.49; *P* = 0.03), without effect modification by sex (Table 2). A strong association of higher FT_4_ with increased risk of cardiovascular mortality was observed in elderly (1.61, 1.19–2.18; *P* = 0.002) (Fig. 2c, d). This association was absent in younger participants (1.03, 0.78–1.34; *P* = 0.85) (Table 3). No association between FT_3_ and cardiovascular mortality was detected (0.91, 0.70–1.18; *P* = 0.48), and risk estimates neither differed between sexes (Table 2) nor between age strata (Table 3).

Sensitivity analyses showed that exclusion of participants who developed malignancy since the previous screening further increased the effects of thyrotropin in the younger (<72 years) participants (1.34, 1.02–1.77; *P* = 0.04) and elderly (≥72 years) (0.68, 0.48–0.97; *P* = 0.03) (Supplementary Table 8). The exclusion of participants with preexisting CVD at baseline reinforced the association of higher FT_4_ and cardiovascular mortality in elderly (≥72 years) (1.73, 1.16–2.57; *P* = 0.007) (Supplementary Table 10). Other sensitivity analyses on the associations between thyrotropin and thyroid function parameters with cardiovascular mortality provided similar results (Supplementary Tables 3–13).

## Discussion

In this analysis of 6,054 individuals from a prospective population-based cohort, we studied potential associations of thyrotropin and thyroid function parameters with all-cause and cardiovascular mortality within the reference ranges of normal thyrotropin and thyroid function. No association was detected between thyrotropin and all-cause mortality. Higher FT_4_ was associated with increased risk of all-cause mortality and was stronger in females than in males. Higher FT_3_ was associated with mortality in females but not in males. Although we could not demonstrate an association between thyrotropin and risk of cardiovascular mortality in the total population, age-stratified analyses unveiled a diverging trend toward increased risk of cardiovascular mortality in younger participants and a reduced risk in elderly with higher thyrotropin. Higher FT_4_ was strongly associated with increased cardiovascular mortality in elderly, independent of traditional cardiovascular risk factors, preexisting CVD, and abnormal thyroid function tests.

Our results agree with accumulating evidence suggesting that a high-normal thyroid function is associated with an increased risk of mortality [5, 7, 8, 15, 16]. They extend previous literature by demonstrating that variations within the reference ranges of normal thyroid function increase the risk of cardiovascular death in elderly individuals. This finding reinforces the need for the revision of the current reference ranges of thyrotropin and FT_4_ according to age and sex and is in favor of an upward shift of thyrotropin and a downward shift of FT_4_ reference ranges. This suggestion is strongly corroborated by a recent study, which suggested that the reference interval for thyrotropin should be shifted upward in older men, whereas the reference interval for FT4 should be compressed compared with conventional reference ranges [23].

More than 90 years ago, Robertson was first to observe reduced longevity in mice fed desiccated thyroid [24]. Subsequent research showed that hypothyroidism considerably prolonged lifespan in Wistar rats compared with their euthyroid counterparts [25], whereas a chronic state of hyperthyroidism initiated at young age and maintained until 12 months of age shortened it [26]. Interestingly, if the state of hyperthyroidism was initiated at young age and maintained until 22 months of age, there seemed to be little, if any, additional adverse effect on lifespan, while initiation of hyperthyroidism at 26 months of age was without effect on further lifespan [27]. We found that the association of FT4 with mortality is modified by age, with strongest associations at higher age. Together, these observations provoke the hypothesis that there is a difference between effects of administered thyroid hormone and of endogenously synthesized thyroid hormone on the process of ageing, and that beyond a certain age effects of endogenously synthesized thyroid hormone may start to mimic that of administered thyroid hormone. In this line of reasoning, it is of interest that the dwarf mice strains Ames and Snell — which show undetectable levels of thyrotropin, growth hormone, and prolactin due to genetic mutations — have an extended lifespan up to 70% and 50% compared with their wild-type age-matched littermates, respectively [27]. This suggests that overactivity of the thyroid gland could adversely affect human longevity as well. Indeed, several studies on thyroid dysfunction have demonstrated that low serum thyrotropin and high FT_4_ are associated with decreased human longevity [12–15] and this effect appears to extend into the high–normal range of thyroid function [5, 7, 8, 15, 16]. Recently, Bano et al. corroborated this finding by showing that middle-aged and elderly individuals with low–normal thyroid function live up to 3.5 years longer than those with a high–normal thyroid function [5].

The observation that high–normal thyroid function is associated with all-cause and cardiovascular mortality in advanced age suggests a subtle and cumulative effect of higher FT_4_. The intrinsic liability of aerobic cellular respiration to produce hazardous by-products, namely reactive oxygen species (ROS), has prompted cells across all domains of mammalian life to evolve intricate antioxidant defense systems balancing the production and clearance of ROS to preserve homeostasis [28]. Although thyroid hormones greatly facilitate aerobic cellular respiration by stimulating mitochondrial biogenesis and promoting oxidative phosphorylation [29], paradoxically, they cause a concomitant net decline of diverse components of the antioxidant defense [30], promoting hyperthyroid individuals to shift into a state of oxidative stress. Indeed, hyperthyroidism both increases ROS production and associated damage to biological macromolecules [29, 30]. We hypothesize that the observed age-related increased cardiovascular mortality risk due to high–normal thyroid function is explained by the qualitative and quantitative decline in antioxidant defense [31].

An inverse relationship between thyrotropin and FT_4_ is anticipated based on the concept of negative feedback regulation. This phenomenon was reflected by our results, which showed that both low–normal thyrotropin and high–normal FT_4_ levels were associated with increased risk of cardiovascular death, with larger risk estimates for FT_4_ than for thyrotropin. Despite its consistency with previous literature [5–7, 15], the latter finding is rather counterintuitive given that thyrotropin is deemed the most sensitive marker for changes in thyroid function [32], because pituitary secretion of thyrotropin is exquisitely sensitive to minute fluctuations in FT_4_ [33]. However, it was recently postulated that FT_4_ levels sensed by the pituitary gland as appropriate could well be inappropriate for the cardiovascular system [17] and potentially account for the observed discrepancy between risk estimates.

Although T_3_ is the prime mediator of thyroid function in cardiovascular physiology [10], we did not observe meaningful associations between FT_3_ and all-cause or cardiovascular mortality. Data on associations between FT_3_ and clinical outcomes within normal ranges of thyroid function are scarce in the literature, complicating a reliable comparison with findings from independent experts. One multicentre, population-based cohort study examining effects of total T_3_ concentrations on the incidence of atrial fibrillation, coronary heart disease, heart failure, hip fracture, dementia, and all-cause mortality did not detect any significant association [6]. Another prospective cohort study did not find any differences in cardiovascular parameters according to quartiles of FT_3_ [16]. Possible explanations could be inadequacy of serum FT_3_ concentrations as a surrogate of tissue T_3_ concentrations or the short half-life of T_3_.

In this general population-based cohort study, with available data on thyroid hormones, we quantified the associations of thyrotropin and the thyroid function parameters FT_4_ and FT_3_ with incidence of all-cause and cardiovascular mortality. We built on previous studies by investigating potential effect modification by sex and age and evaluated the influence of abnormal thyroid function tests on these associations. We applied consistent methodology throughout the analyses to diminish residual confounding and potential reverse causality and ascertained the robustness of our findings by conducting an extensive range of sensitivity analyses. A noteworthy strength was the size of the study, the elaborate and complete follow-up, and the wide range of the participants’ ages, allowing us to retain statistical power while restricting the analyses, assess potential time-dependent effects of thyrotropin and thyroid function parameters on all-cause and cardiovascular mortality across time, and investigate potential effect modification by age and sex.

Our samples had been stored for 14–17 years at −80 °C and had not been thawed before. This time of storage before assessment is comparable to that of other prospective studies, such as the Malmö Diet and Cancer study, wherein samples had been stored for 14–19 years and were subjected to two freeze-thaw cycles before measurements were performed [34]. They also considered the validity of such samples. It was first evaluated whether frozen samples differ from fresh samples. This has been investigated by Männistö et al. who found that, thyrotropin, FT_4_, and antithyroid peroxidase levels were very similar when analyzed in frozen versus fresh serum samples. For FT_3_, however, levels were slightly higher in frozen/thawed samples as compared with fresh serum samples [35]. Concerning storage time, the same authors reported that thyrotropin, FT_4_, and FT_3_ levels were not affected by storage for 14–18 years, but antithyroid peroxidase levels were only stable for up to 14 years, following which they displayed a slight increase [36]. Thus, freezing/thawing and storage time may have affected the absolute levels of FT_3_ and antithyroid peroxidase in our study, but there is nothing to suggest that effects of freezing/thawing and storage time on samples differed between subjects that developed an event and subjects that did not develop an event, making it unlikely that relative comparisons such as risk estimates could have been affected. The evaluation of thyrotropin and thyroid function parameters was based on a single measurement and within-person variation may therefore be a potential source for measurement error. In many instances, hormonal measurements were done more than a decade before the study outcomes, so it was not really known what the thyroid status of study participants were at the time of the events. Importantly, due to the phenomenon of regression dilution bias, the association between variables assessed at one time point — like thyroid function tests in this case — and prospective development of events, is underestimated [37]. Indeed, if we would have had data of repeated measurements on thyroid function tests, this would likely have resulted in strengthening of associations to be found, like previously has been shown for thyrotropin and FT_4_ [37]. In addition, our study almost exclusively included Caucasians, requiring verification of our findings in other populations. Lastly, because of the observational nature of our study, we cannot conclude that positive associations between high–normal thyroid function and cardiovascular mortality in elderly reflect cause and effect relations nor can we entirely eliminate the possibility of residual confounding.

In conclusion, the findings from this prospective cohort study showed that elderly individuals from the general population with a high–normal FT_4_ are at increased risk of all-cause and cardiovascular death and emphasize the need of redefining the current reference ranges of thyroid function according to age and sex. Further studies are warranted to replicate our findings and shed light on the mechanistic basis for the differences in cardiovascular mortality risk within reference ranges of normal thyroid function.

## Supplementary information

Supplementary Online Content
