# Supplementary material for: Thyroid function and risk of all-cause and cardiovascular mortality: a prospective population-based cohort study
Source: Endocrine. 2020 Jul 6;71(2):385–96. doi: 10.1007/s12020-020-02397-z (PMC7881952; doi:10.1007/s12020-020-02397-z)
Supplement: Supplementary file 1 — Supplementary Online Content [file 12020_2020_2397_MOESM1_ESM.docx]

**Supplementary Online Content**

Groothof D, Flores-Guerrero, JL, Nolte IM, et al. Thyroid function and risk of all-cause and cardiovascular mortality: a prospective population-based cohort study

**Supplementary Table 1.** Percentage of missing values per covariate to be imputed

**Supplementary Table 2.** Total and age-specific correlations between thyrotropin and thyroid hormones

**Supplementary Table 3.** Total and sex-stratified prospective associations of thyrotropin and thyroid function with all-cause and cardiovascular mortality after exclusion of 512 (8.3%) participants with a positive anti-thyroid peroxidase titer

**Supplementary Table 4**. Total and age-stratified prospective associations of thyrotropin and thyroid function with all-cause and cardiovascular mortality after exclusion of 512 (8.3%) participants with a positive anti-thyroid peroxidase titer

**Supplementary Table 5.** Total and sex-stratified prospective associations of thyrotropin and thyroid function with all-cause and cardiovascular mortality after exclusion of 221 (3.6%) participants with FT_4_ and/or FT_3_ outside the reference ranges

**Supplementary Table 6.** Total and age-stratified prospective associations of thyrotropin and thyroid function with all-cause and cardiovascular mortality after exclusion of 221 (3.6%) participants with FT_4_ and/or FT_3_ outside the reference ranges

**Supplementary Table 7.** Total and sex-stratified prospective associations of thyrotropin and thyroid function with all-cause and cardiovascular mortality after exclusion of 276 (4.5%) participants with incident malignancy since the previous screening

**Supplementary Table 8.** Total and age-stratified prospective associations of thyrotropin and thyroid function with all-cause and cardiovascular mortality after exclusion of 276 (4.5%) participants with incident malignancy since the previous screening

**Supplementary Table 9.** Total and sex-stratified prospective associations of thyrotropin and thyroid function with all-cause and cardiovascular mortality after exclusion of 389 (6.4%) participants with pre-existing cardiovascular disease at baseline

**Supplementary Table 10.** Total and age-stratified prospective associations of thyrotropin and thyroid function with all-cause and cardiovascular mortality after exclusion of 389 (6.4%) participants with pre-existing cardiovascular disease at baseline

**Supplementary Table 11.** Total and sex-stratified prospective associations of thyrotropin and thyroid function with all-cause and cardiovascular mortality after exclusion of 51 (13.4%) deaths from all-causes and 20 (19.4%) from cardiovascular disease during the first two years of follow-up

**Supplementary Table 12.** Total and age-stratified prospective associations of thyrotropin and thyroid function with all-cause and cardiovascular mortality after exclusion of 51 (13.4%) deaths from all-causes and 20 (19.4%) from cardiovascular disease during the first two years of follow-up

**Supplementary Table 13.** Total and age-stratified prospective associations of thyrotropin and thyroid function with all-cause and cardiovascular mortality after stratification by the age of 65 years

| **Supplementary Table 1: Percentage of missing values per covariate to be imputed** | | |
| --- | --- | --- |
| **Sociodemographic characteristics** | | **Percentage of missing values** |
|  | Age | 0.0 |
|  | Sex | 0.0 |
|  | Race | 0.0 |
|  | Education | 0.0 |
|  | Current smoking | 1.3 |
|  | Drinking alcohol | 1.0 |
|  | Type 2 diabetes | 0.5 |
|  | History of CVD | 0.0 |
| **Body composition** | |  |
|  | BMI | 0.0 |
| **Haemodynamics** | |  |
|  | SBP | 0.1 |
|  | DBP | 0.1 |
| **Lipid spectrum** | |  |
|  | Total cholesterol | 0.6 |
|  | HDL cholesterol | 3.1 |
|  | Triglycerides | 3.1 |
| **Inflammation** | |  |
|  | Hs-CRP | 16.6 |
| **Kidney function parameters** | |  |
|  | eGFR_SCr-CysC_ | 4.7 |
|  | UAE | 0.5 |
| **Medication** | |  |
|  | Antihypertensive drugs | 0.0 |
|  | Lipid-lowering drugs | 0.0 |
| Abbreviations: BMI, body-mass index; CVD, cardiovascular disease; CysC, serum cystatin C; DBP, diastolic blood pressure; eGFR, estimated glomerular filtration rate; HDL, high-density lipoprotein; Hs-CRP, high-sensitivity C-reactive protein; SCr, serum creatinine; UAE, urinary albumin excretion. | | |

| **Supplementary Table 2.** Total and age-specific correlations between thyrotropin and thyroid hormones | | | | |
| --- | --- | --- | --- | --- |
|  |  | **All participants: *N* = 6,164** | | |
|  |  |  | | |
| **Markers** |  | Thyrotropin | FT_4_ | FT_3_ |
| Thyrotropin |  | 1 |  |  |
| FT_4_ |  | –0.16 (–0.18 to –0.13) | 1 |  |
| FT_3_ |  | –0.05 (–0.08 to –0.03) | 0.23 (0.20 to 0.25) | 1 |
|  |  |  |  |  |
|  |  | **Younger participants (age ≤72 years), *n* = 5,612** | | |
|  |  |  | | |
| **Markers** |  | Thyrotropin | FT_4_ | FT_3_ |
| Thyrotropin |  | 1 |  |  |
| FT_4_ |  | –0.15 (–0.18 to –0.12) | 1 |  |
| FT_3_ |  | –0.05 (–0.08 to –0.03) | 0.25 (0.22 to 0.27) | 1 |
|  |  |  |  |  |
|  |  | **Elderly participants (age >72 years), *n* = 534** | | |
|  |  |  | | |
| **Markers** |  | Thyrotropin | FT_4_ | FT_3_ |
| Thyrotropin |  | 1 |  |  |
| FT_4_ |  | –0.22 (–0.30 to –0.14) | 1 |  |
| FT_3_ |  | –0.10 (–0.18 to –0.01) | 0.09 (0.00 to 0.17) | 1 |
|  |  |  |  |  |
| Abbreviations: FT_3_, free triiodothyronine. FT_4_, free thyroxine.  *Pearson correlation parameters are given as *ρ* (95% CI). | | | | |

| Supplementary Table 3: Total and sex-stratified prospective associations of thyrotropin and thyroid function with all-cause and cardiovascular mortality after exclusion of 512 (8.3%) participants with a positive anti-thyroid peroxidase titer | | | | | | | | | | | | | |
| --- | --- | --- | --- | --- | --- | --- | --- | --- | --- | --- | --- | --- | --- |
|  | | | | | | | | | | | | | |
|  |  |  | **TSH** | | |  | **FT_4_** | | |  | **FT_3_** | | |
| All-cause mortality | |  | **HR (95% CI)** | ***P* value** | ***P*_interaction_^a^** |  | **HR (95% CI)** | ***P* value** | ***P*_interaction_^a^** |  | **HR (95% CI)** | ***P* value** | ***P*_interaction_^a^** |
| Total population  n_events_/n_total_ = 353/5579 | |  |  |  |  |  |  |  |  |  |  |  |  |
|  | Crude |  | 0.96 (0.86-1.07) | .42 |  |  | 1.29 (1.16-1.43) | <.001 |  |  | 0.84 (0.74-0.96) | .01 |  |
|  | Age- and sex-adjusted |  | 1.01 (0.91-1.13) | .82 | .70 |  | 1.24 (1.12-1.37) | <.001 | .03 |  | 0.97 (0.86-1.09) | .57 | .03 |
|  | Multivariable model^b^ |  | 1.05 (0.94-1.17) | .42 | .77 |  | 1.17 (1.05-1.29) | .003 | .01 |  | 0.97 (0.86-1.09) | .59 | .08 |
| Men  n_events_/n_total_ = 273/3010 | |  |  |  |  |  |  |  |  |  |  |  |  |
|  | Crude |  | 0.96 (0.84-1.10) | .55 |  |  | 1.11 (0.99-1.25) | .07 |  |  | 0.60 (0.51-0.70) | <.001 |  |
|  | Age-adjusted |  | 1.00 (0.88-1.14) | .97 |  |  | 1.17 (1.04-1.31) | .008 |  |  | 0.92 (0.79-1.08) | .32 |  |
|  | Multivariable model^c^ |  | 1.00 (0.88-1.14) | .95 |  |  | 1.13 (1.01-1.27) | .04 |  |  | 0.93 (0.80-1.08) | .34 |  |
| Women  n_events_/n_total_ = 80/2569 | |  |  |  |  |  |  |  |  |  |  |  |  |
|  | Crude |  | 1.07 (0.87-1.33) | .51 |  |  | 1.70 (1.37-2.11) | <.001 |  |  | 1.07 (0.91-1.26) | .43 |  |
|  | Age-adjusted |  | 1.06 (0.86-1.30) | .61 |  |  | 1.57 (1.27-1.95) | <.001 |  |  | 1.14 (0.97-1.33) | .12 |  |
|  | Multivariable model^c^ |  | 1.15 (0.93-1.42) | .20 |  |  | 1.49 (1.18-1.87) | .001 |  |  | 1.11 (0.91-1.36) | .30 |  |
| Cardiovascular mortality | |  | **HR (95% CI)** | ***P* value** | ***P*_interaction_^a^** |  | **HR (95% CI)** | ***P* value** | ***P*_interaction_^a^** |  | **HR (95% CI)** | ***P* value** | ***P*_interaction_^a^** |
| Total population  n_events_/n_total_ = 99/5579 | |  |  |  |  |  |  |  |  |  |  |  |  |
|  | Crude |  | 0.91 (0.74-1.12) | .38 |  |  | 1.40 (1.16-1.70) | <.001 |  |  | 0.68 (0.53-0.88) | .004 |  |
|  | Age- and sex-adjusted |  | 0.98 (0.80-1.20) | .82 | .64 |  | 1.34 (1.11-1.62) | .003 | .55 |  | 0.83 (0.63-1.09) | .18 | .72 |
|  | Multivariable model^b^ |  | 0.98 (0.79-1.22) | .86 | .61 |  | 1.22 (1.01-1.47) | .04 | .32 |  | 0.87 (0.66-1.14) | .32 | .96 |
| Men  n_events_/n_total_ = 80/3010 | |  |  |  |  |  |  |  |  |  |  |  |  |
|  | Crude |  | 0.90 (0.71-1.16) | .42 |  |  | 1.24 (1.00-1.53) | .05 |  |  | 0.49 (0.36-0.66) | <.001 |  |
|  | Age-adjusted |  | 0.95 (0.75-1.21) | .69 |  |  | 1.30 (1.05-1.61) | .02 |  |  | 0.82 (0.60-1.12) | .20 |  |
|  | Multivariable model^c^ |  | 0.91 (0.71-1.17) | .46 |  |  | 1.23 (1.00-1.52) | .06 |  |  | 0.85 (0.62-1.16) | .30 |  |
| Women  n_events_/n_total_ = 19/2569 | |  |  |  |  |  |  |  |  |  |  |  |  |
|  | Crude |  | 1.10 (0.71-1.70) | .68 |  |  | 1.77 (1.14-2.73) | .01 |  |  | 0.81 (0.44-1.49) | .50 |  |
|  | Age-adjusted |  | 1.08 (0.71-1.64) | .73 |  |  | 1.54 (1.01-2.34) | .005 |  |  | 0.90 (0.49-1.65) | .74 |  |
|  | Multivariable model^c^ |  | 1.22 (0.78-1.91) | .39 |  |  | 1.39 (0.86-2.26) | .17 |  |  | 0.87 (0.48-1.55) | .63 |  |
| Abbreviations: CI, confidence interval; FT_3_, free triiodothyronine; FT_4_, free thyroxine; HR, hazard ratio; TSH, thyrotropin.  Point estimates are expressed per SD increment in the predictor.  ^a^Evidence against the null hypothesis of no effect modification by sex on the association of the thyroid function parameter and the specific outcome concerned.  ^b^Adjusted for age, sex, current smoking, body-mass index, systolic blood pressure, use of antihypertensive drugs, total cholesterol/high-density lipoprotein ratio, ln triglycerides, use of lipid-lowering drugs, type 2 diabetes, high-sensitive C-reactive protein, history of cardiovascular disease, estimated glomerular filtration rate, and ln urinary albumin excretion.  ^c^Adjusted for all potential confounders except for sex. | | | | | | | | | | | | | |

| Supplementary Table 4: Total and age-stratified prospective associations of thyrotropin and thyroid function with all-cause and cardiovascular mortality after exclusion of 512 (8.3%) participants with a positive anti-thyroid peroxidase titer | | | | | | | | | | | | | |
| --- | --- | --- | --- | --- | --- | --- | --- | --- | --- | --- | --- | --- | --- |
|  | | | | | | | | | | | | | |
|  |  |  | **TSH** | | |  | **FT_4_** | | |  | **FT_3_** | | |
| All-cause mortality | |  | **HR (95% CI)** | ***P* value** | ***P*_interaction_^a^** |  | **HR (95% CI)** | ***P* value** | ***P*_interaction_^a^** |  | **HR (95% CI)** | ***P* value** | ***P*_interaction_^a^** |
| Total population  n_events_/n_total_ = 353/5579 | |  |  |  |  |  |  |  |  |  |  |  |  |
|  | Crude |  | 0.96 (0.86-1.07) | .42 |  |  | 1.29 (1.16-1.43) | <.001 |  |  | 0.84 (0.74-0.96) | .01 |  |
|  | Age- and sex-adjusted |  | 1.01 (0.91-1.13) | .82 | .36 |  | 1.24 (1.12-1.37) | <.001 | .16 |  | 0.97 (0.86-1.09) | .57 | .53 |
|  | Multivariable model^b^ |  | 1.05 (0.94-1.17) | .42 | .67 |  | 1.17 (1.05-1.29) | .003 | .39 |  | 0.97 (0.86-1.09) | .59 | .55 |
| Low age (<70 years)^c^  n_events_/n_total_ = 176/4888 | |  |  |  |  |  |  |  |  |  |  |  |  |
|  | Crude |  | 0.88 (0.75-1.03) | .11 |  |  | 1.14 (0.98-1.33) | .08 |  |  | 0.95 (0.80-1.13) | .58 |  |
|  | Age- and sex-adjusted |  | 0.91 (0.77-1.06) | .23 |  |  | 1.13 (0.98-1.30) | .10 |  |  | 0.95 (0.80-1.12) | .54 |  |
|  | Multivariable model^b^ |  | 1.01 (0.86-1.19) | .88 |  |  | 1.08 (0.94-1.26) | .28 |  |  | 0.96 (0.81-1.12) | .58 |  |
| High age (≥70 years)^c^  n_events_/n_total_ = 177/691 | |  |  |  |  |  |  |  |  |  |  |  |  |
|  | Crude |  | 1.08 (0.93-1.24) | .32 |  |  | 1.36 (1.18-1.57) | <.001 |  |  | 1.03 (0.85-1.26) | .76 |  |
|  | Age- and sex-adjusted |  | 1.12 (0.97-1.29) | .13 |  |  | 1.37 (1.19-1.59) | <.001 |  |  | 1.02 (0.83-1.24) | .88 |  |
|  | Multivariable model^b^ |  | 1.09 (0.94-1.27) | .26 |  |  | 1.30 (1.12-1.51) | .001 |  |  | 1.03 (0.85-1.26) | .75 |  |
| Cardiovascular mortality | |  | **HR (95% CI)** | ***P* value** | ***P*_interaction_^a^** |  | **HR (95% CI)** | ***P* value** | ***P*_interaction_^a^** |  | **HR (95% CI)** | ***P* value** | ***P*_interaction_^a^** |
| Total population  n_events_/n_total_ = 99/5579 | |  |  |  |  |  |  |  |  |  |  |  |  |
|  | Crude |  | 0.91 (0.74-1.12) | .38 |  |  | 1.40 (1.16-1.70) | <.001 |  |  | 0.68 (0.53-0.88) | .004 |  |
|  | Age- and sex-adjusted |  | 0.98 (0.80-1.20) | .82 | .10 |  | 1.34 (1.11-1.62) | <.001 | .03 |  | 0.83 (0.63-1.09) | .18 | .23 |
|  | Multivariable model^b^ |  | 0.98 (0.79-1.22) | .86 | .03 |  | 1.22 (1.01-1.47) | .04 | .05 |  | 0.87 (0.66-1.14) | .32 | .34 |
| Low age (<72 years)^c^  n_events_/n_total_ = 49/5113 | |  |  |  |  |  |  |  |  |  |  |  |  |
|  | Crude |  | 1.07 (0.80-1.42) | .66 |  |  | 1.19 (0.90-1.57) | .23 |  |  | 0.92 (0.66-1.30) | .64 |  |
|  | Age- and sex-adjusted |  | 1.10 (0.84-1.45) | .48 |  |  | 1.14 (0.88-1.48) | .32 |  |  | 0.95 (0.70-1.31) | .77 |  |
|  | Multivariable model^b^ |  | 1.31 (0.98-1.75) | .07 |  |  | 1.00 (0.76-1.31) | >.99 |  |  | 0.96 (0.69-1.34) | .82 |  |
| High age (≥72 years)^c^  n_events_/n_total_ = 50/466 | |  |  |  |  |  |  |  |  |  |  |  |  |
|  | Crude |  | 0.83 (0.61-1.13) | .23 |  |  | 1.56 (1.18-2.06) | .002 |  |  | 0.72 (0.46-1.09) | .12 |  |
|  | Age- and sex-adjusted |  | 0.85 (0.62-1.16) | .31 |  |  | 1.64 (1.22-2.20) | .001 |  |  | 0.70 (0.46-1.06) | .09 |  |
|  | Multivariable model^b^ |  | 0.78 (0.56-1.09) | .15 |  |  | 1.67 (1.22-2.30) | .001 |  |  | 0.78 (0.52-1.18) | .25 |  |
| Abbreviations: CI, confidence interval; FT_3_, free triiodothyronine; FT_4_, free thyroxine; HR, hazard ratio; TSH, thyrotropin.  Point estimates are expressed per SD increment in the predictor.  ^a^Evidence against the null hypothesis of no effect modification by age on the association of the thyroid function parameter and the specific outcome concerned.  ^b^Adjusted for age, sex, current smoking, body-mass index, systolic blood pressure, use of antihypertensive drugs, total cholesterol/high-density lipoprotein ratio, ln triglycerides, use of lipid-lowering drugs, type 2 diabetes, high-sensitive C-reactive protein, history of cardiovascular disease, estimated glomerular filtration rate, and ln urinary albumin excretion.  ^c^Cut-off values are established through dichotomisation of the cohort by the median age for half the events, to obtain an equal amount of events per stratum. | | | | | | | | | | | | | |

| Supplementary Table 5: Total and sex-stratified prospective associations of thyrotropin and thyroid function with all-cause and cardiovascular mortality after exclusion of 221 (3.6%) participants with FT_4_ and/or FT_3_ outside the reference ranges | | | | | | | | | | | | | |
| --- | --- | --- | --- | --- | --- | --- | --- | --- | --- | --- | --- | --- | --- |
|  |  |  |  | | |  |  | | |  |  | | |
|  |  |  | **TSH** | | |  | **FT_4_** | | |  | **FT_3_** | | |
| All-cause mortality | |  | **HR (95% CI)** | ***P* value** | ***P*_interaction_^a^** |  | **HR (95% CI)** | ***P* value** | ***P*_interaction_^a^** |  | **HR (95% CI)** | ***P* value** | ***P*_interaction_^a^** |
| Total population  n_events_/n_total_ = 361/5846 | |  |  |  |  |  |  |  |  |  |  |  |  |
|  | Crude |  | 0.95 (0.85-1.05) | .31 |  |  | 1.24 (1.10-1.39) | <.001 |  |  | 0.82 (0.72-0.94) | .005 |  |
|  | Age- and sex-adjusted |  | 1.02 (0.92-1.13) | .75 | .94 |  | 1.20 (1.08-1.35) | .001 | .43 |  | 0.93 (0.81-1.08) | .36 | .03 |
|  | Multivariable model^b^ |  | 1.06 (0.95-1.18) | .31 | .96 |  | 1.15 (1.02-1.30) | .02 | .38 |  | 0.94 (0.81-1.08) | .38 | .09 |
| Men  n_events_/n_total_ = 275/3050 | |  |  |  |  |  |  |  |  |  |  |  |  |
|  | Crude |  | 0.98 (0.86-1.11) | .71 |  |  | 1.10 (0.96-1.25) | .17 |  |  | 0.57 (0.48-0.67) | <.001 |  |
|  | Age-adjusted |  | 1.02 (0.90-1.15) | .79 |  |  | 1.18 (1.03-1.34) | .02 |  |  | 0.88 (0.74-1.05) | .15 |  |
|  | Multivariable model^c^ |  | 1.03 (0.91-1.17) | .68 |  |  | 1.15 (1.01-1.32) | .04 |  |  | 0.89 (0.75-1.06) | .20 |  |
| Women  n_events_/n_total_ = 86/2796 | |  |  |  |  |  |  |  |  |  |  |  |  |
|  | Crude |  | 1.04 (0.85-1.27) | .72 |  |  | 1.43 (1.13-1.81) | .003 |  |  | 1.10 (0.83-1.47) | .50 |  |
|  | Age-adjusted |  | 1.03 (0.84-1.25) | .81 |  |  | 1.35 (1.07-1.70) | .01 |  |  | 1.20 (0.90-1.60) | .20 |  |
|  | Multivariable model^c^ |  | 1.13 (0.93-1.38) | .22 |  |  | 1.21 (0.95-1.55) | .13 |  |  | 1.08 (0.81-1.44) | .59 |  |
| Cardiovascular mortality | |  | **HR (95% CI)** | ***P* value** | ***P*_interaction_^a^** |  | **HR (95% CI)** | ***P* value** | ***P*_interaction_^a^** |  | **HR (95% CI)** | ***P* value** | ***P*_interaction_^a^** |
| Total population  n_events_/n_total_ = 100/5846 | |  |  |  |  |  |  |  |  |  |  |  |  |
|  | Crude |  | 0.91 (0.74-1.12) | .36 |  |  | 1.40 (1.13-1.73) | .002 |  |  | 0.73 (0.56-0.94) | .02 |  |
|  | Age- and sex-adjusted |  | 0.99 (0.81-1.22) | .95 | .92 |  | 1.35 (1.09-1.67) | .007 | .30 |  | 0.87 (0.65-1.15) | .32 | .46 |
|  | Multivariable model^b^ |  | 1.02 (0.83-1.25) | .87 | .94 |  | 1.27 (1.02-1.59) | .03 | .25 |  | 0.92 (0.69-1.22) | .56 | .67 |
| Men  n_events_/n_total_ = 80/3050 | |  |  |  |  |  |  |  |  |  |  |  |  |
|  | Crude |  | 0.94 (0.74-1.20) | .62 |  |  | 1.18 (0.93-1.50) | .17 |  |  | 0.50 (0.37-0.67) | <.001 |  |
|  | Age-adjusted |  | 0.99 (0.79-1.25) | .94 |  |  | 1.27 (1.00-1.62) | .05 |  |  | 0.84 (0.61-1.15) | .27 |  |
|  | Multivariable model^c^ |  | 0.97 (0.76-1.24) | .82 |  |  | 1.24 (0.97-1.60) | .09 |  |  | 0.89 (0.64-1.23) | .48 |  |
| Women  n_events_/n_total_ = 20/2796 | |  |  |  |  |  |  |  |  |  |  |  |  |
|  | Crude |  | 1.02 (0.67-1.56) | .91 |  |  | 1.93 (1.21-3.08) | .006 |  |  | 0.92 (0.51-1.68) | .80 |  |
|  | Age-adjusted |  | 1.01 (0.67-1.53) | .95 |  |  | 1.73 (1.09-2.75) | .02 |  |  | 1.04 (0.57-1.91) | .90 |  |
|  | Multivariable model^c^ |  | 1.18 (0.77-1.82) | .44 |  |  | 1.48 (0.89-2.44) | .13 |  |  | 0.95 (0.53-1.72) | .87 |  |
| Abbreviations: CI, confidence interval; FT_3_, free triiodothyronine; FT_4_, free thyroxine; HR, hazard ratio; TSH, thyrotropin.  Point estimates are expressed per SD increment in the predictor.  ^a^Evidence against the null hypothesis of no effect modification by sex on the association of the thyroid function parameter and the specific outcome concerned.  ^b^Adjusted for age, sex, current smoking, body-mass index, systolic blood pressure, use of antihypertensive drugs, total cholesterol/high-density lipoprotein ratio, ln triglycerides, use of lipid-lowering drugs, type 2 diabetes, high-sensitive C-reactive protein, history of cardiovascular disease, estimated glomerular filtration rate, and ln urinary albumin excretion.  ^c^Adjusted for all potential confounders except for sex. | | | | | | | | | | | | | |

| Supplementary Table 6: Total and age-stratified prospective associations of thyrotropin and thyroid function with all-cause and cardiovascular mortality after exclusion of 221 (3.6%) participants with FT_4_ and/or FT_3_ outside the reference ranges | | | | | | | | | | | | | |
| --- | --- | --- | --- | --- | --- | --- | --- | --- | --- | --- | --- | --- | --- |
|  | | | | | | | | | | | | | |
|  |  |  | **TSH** | | |  | **FT_4_** | | |  | **FT_3_** | | |
| All-cause mortality | |  | **HR (95% CI)** | ***P* value** | ***P*_interaction_^a^** |  | **HR (95% CI)** | ***P* value** | ***P*_interaction_^a^** |  | **HR (95% CI)** | ***P* value** | ***P*_interaction_^a^** |
| Total population  n_events_/n_total_ = 361/5846 | |  |  |  |  |  |  |  |  |  |  |  |  |
|  | Crude |  | 0.95 (0.85-1.05) | .31 |  |  | 1.24 (1.10-1.39) | <.001 |  |  | 0.82 (0.72-0.94) | .005 |  |
|  | Age- and sex-adjusted |  | 1.02 (0.92-1.13) | .75 | .54 |  | 1.20 (1.08-1.35) | .001 | .44 |  | 0.93 (0.81-1.08) | .36 | .60 |
|  | Multivariable model^b^ |  | 1.06 (0.95-1.18) | .31 | .79 |  | 1.15 (1.02-1.30) | .02 | .74 |  | 0.94 (0.81-1.08) | .38 | .66 |
| Low age (<70 years)^c^  n_events_/n_total_ = 180/5113 | |  |  |  |  |  |  |  |  |  |  |  |  |
|  | Crude |  | 0.88 (0.76-1.03) | .12 |  |  | 1.07 (0.91-1.26) | .42 |  |  | 1.00 (0.83-1.20) | .99 |  |
|  | Age- and sex-adjusted |  | 0.92 (0.79-1.07) | .29 |  |  | 1.09 (0.92-1.28) | .33 |  |  | 0.99 (0.81-1.21) | .94 |  |
|  | Multivariable model^b^ |  | 1.03 (0.88-1.20) | .75 |  |  | 1.07 (0.91-1.27) | .41 |  |  | 0.98 (0.80-1.21) | .88 |  |
| High age (≥70 years)^c^  n_events_/n_total_ = 181/733 | |  |  |  |  |  |  |  |  |  |  |  |  |
|  | Crude |  | 1.05 (0.91-1.20) | .54 |  |  | 1.35 (1.15-1.58) | <.001 |  |  | 0.96 (0.78-1.18) | .69 |  |
|  | Age- and sex-adjusted |  | 1.12 (0.97-1.29) | .13 |  |  | 1.34 (1.14-1.58) | <.001 |  |  | 0.89 (0.72-1.11) | .31 |  |
|  | Multivariable model^b^ |  | 1.11 (0.96-1.28) | .17 |  |  | 1.26 (1.06-1.49) | .007 |  |  | 0.93 (0.75-1.15) | .49 |  |
| Cardiovascular mortality | |  | **HR (95% CI)** | ***P* value** | ***P*_interaction_^a^** |  | **HR (95% CI)** | ***P* value** | ***P*_interaction_^a^** |  | **HR (95% CI)** | ***P* value** | ***P*_interaction_^a^** |
| Total population  n_events_/n_total_ = 100/5846 | |  |  |  |  |  |  |  |  |  |  |  |  |
|  | Crude |  | 0.91 (0.74-1.12) | .36 |  |  | 1.40 (1.13-1.73) | .002 |  |  | 0.73 (0.56-0.94) | .02 |  |
|  | Age- and sex-adjusted |  | 0.99 (0.81-1.22) | .95 | .08 |  | 1.35 (1.09-1.67) | .007 | .13 |  | 0.87 (0.65-1.15) | .32 | .09 |
|  | Multivariable model^b^ |  | 1.02 (0.83-1.25) | .87 | .04 |  | 1.27 (1.02-1.59) | .03 | .21 |  | 0.92 (0.69-1.22) | .56 | .14 |
| Low age (<72 years)^c^  n_events_/n_total_ = 50/5365 | |  |  |  |  |  |  |  |  |  |  |  |  |
|  | Crude |  | 1.07 (0.81-1.40) | .65 |  |  | 1.16 (0.85-1.57) | .35 |  |  | 1.03 (0.72-1.46) | .88 |  |
|  | Age- and sex-adjusted |  | 1.11 (0.86-1.45) | .42 |  |  | 1.15 (0.85-1.55) | .36 |  |  | 1.07 (0.73-1.55) | .74 |  |
|  | Multivariable model^b^ |  | 1.31 (1.00-1.71) | .05 |  |  | 1.10 (0.80-1.53) | .55 |  |  | 1.09 (0.74-1.62) | .66 |  |
| High age (≥72 years)^c^  n_events_/n_total_ = 51/490 | |  |  |  |  |  |  |  |  |  |  |  |  |
|  | Crude |  | 0.84 (0.62-1.14) | .26 |  |  | 1.56 (1.15-2.12) | .005 |  |  | 0.72 (0.47-1.11) | .14 |  |
|  | Age- and sex-adjusted |  | 0.86 (0.63-1.18) | .36 |  |  | 1.60 (1.17-2.20) | .004 |  |  | 0.68 (0.44-1.05) | .08 |  |
|  | Multivariable model^b^ |  | 0.81 (0.58-1.13) | .22 |  |  | 1.60 (1.14-2.23) | .006 |  |  | 0.76 (0.50-1.17) | .21 |  |
| Abbreviations: CI, confidence interval; FT_3_, free triiodothyronine; FT_4_, free thyroxine; HR, hazard ratio; TSH, thyrotropin.  Point estimates are expressed per SD increment in the predictor.  ^a^Evidence against the null hypothesis of no effect modification by age on the association of the thyroid function parameter and the specific outcome concerned.  ^b^Adjusted for age, sex, current smoking, body-mass index, systolic blood pressure, use of antihypertensive drugs, total cholesterol/high-density lipoprotein ratio, ln triglycerides, use of lipid-lowering drugs, type 2 diabetes, high-sensitive C-reactive protein, history of cardiovascular disease, estimated glomerular filtration rate, and ln urinary albumin excretion.  ^c^Cut-off values are established through dichotomisation of the cohort by the median age for half the events, to obtain an equal amount of events per stratum. | | | | | | | | | | | | | |

| Supplementary Table 7: Total and sex-stratified prospective associations of thyrotropin and thyroid function with all-cause and cardiovascular mortality after exclusion of 276 (4.5%) participants with incident malignancy since the previous screening | | | | | | | | | | | | | | | |
| --- | --- | --- | --- | --- | --- | --- | --- | --- | --- | --- | --- | --- | --- | --- | --- |
|  | | | | | | | | | | | | | | | |
|  |  |  | **TSH** | | |  | **FT_4_** | | |  | | **FT_3_** | | | |
| All-cause mortality | |  | **HR (95% CI)** | ***P* value** | ***P*_interaction_^a^** |  | **HR (95% CI)** | ***P* value** | ***P*_interaction_^a^** | |  | | **HR (95% CI)** | ***P* value** | ***P*_interaction_^a^** |
| Total population  n_events_/n_total_ = 345/5789 | |  |  |  |  |  |  |  |  | |  | |  |  |  |
|  | Crude |  | 0.93 (0.83-1.04) | .18 |  |  | 1.29 (1.16-1.43) | <.001 |  | |  | | 0.89 (0.78-1.02) | .09 |  |
|  | Age- and sex-adjusted |  | 0.99 (0.89-1.10) | .85 | .94 |  | 1.23 (1.11-1.37) | <.001 | .03 | |  | | 1.01 (0.88-1.16) | .88 | .01 |
|  | Multivariable model^b^ |  | 1.02 (0.91-1.14) | .73 | .97 |  | 1.17 (1.06-1.30) | .003 | .009 | |  | | 1.02 (0.89-1.17) | .81 | .04 |
| Men  n_events_/n_total_ = 263/2993 | |  |  |  |  |  |  |  |  | |  | |  |  |  |
|  | Crude |  | 0.95 (0.84-1.09) | .48 |  |  | 1.11 (0.98-1.25) | .10 |  | |  | | 0.62 (0.52-0.72) | <.001 |  |
|  | Age-adjusted |  | 0.99 (0.87-1.12) | .87 |  |  | 1.16 (1.03-1.30) | .02 |  | |  | | 0.94 (0.80-1.11) | .46 |  |
|  | Multivariable model^c^ |  | 0.99 (0.87-1.13) | .93 |  |  | 1.13 (1.00-1.27) | .05 |  | |  | | 0.95 (0.81-1.12) | .55 |  |
| Women  n_events_/n_total_ = 82/2796 | |  |  |  |  |  |  |  |  | |  | |  |  |  |
|  | Crude |  | 1.02 (0.83-1.26) | .84 |  |  | 1.69 (1.36-2.09) | <.001 |  | |  | | 1.10 (0.96-1.25) | .18 |  |
|  | Age-adjusted |  | 1.00 (0.82-1.23) | .98 |  |  | 1.57 (1.28-1.94) | <.001 |  | |  | | 1.16 (1.02-1.33) | .02 |  |
|  | Multivariable model^c^ |  | 1.09 (0.89-1.34) | .41 |  |  | 1.47 (1.18-1.84) | .001 |  | |  | | 1.16 (0.98-1.37) | .09 |  |
| Cardiovascular mortality | |  | **HR (95% CI)** | ***P* value** | ***P*_interaction_^a^** |  | **HR (95% CI)** | ***P* value** | ***P*_interaction_^a^** | |  | | **HR (95% CI)** | ***P* value** | ***P*_interaction_^a^** |
| Total population  n_events_/n_total_ = 96/5789 | |  |  |  |  |  |  |  |  | |  | |  |  |  |
|  | Crude |  | 0.89 (0.73-1.10) | .30 |  |  | 1.33 (1.09-1.62) | .004 |  | |  | | 0.74 (0.57-0.96) | .02 |  |
|  | Age- and sex-adjusted |  | 0.97 (0.79-1.19) | .78 | .91 |  | 1.26 (1.04-1.52) | .02 | .33 | |  | | 0.87 (0.66-1.14) | .31 | .61 |
|  | Multivariable model^b^ |  | 0.97 (0.78-1.20) | .75 | .85 |  | 1.17 (0.97-1.43) | .10 | .20 | |  | | 0.92 (0.70-1.22) | .57 | .87 |
| Men  n_events_/n_total_ = 78/2993 | |  |  |  |  |  |  |  |  | |  | |  |  |  |
|  | Crude |  | 0.92 (0.72-1.18) | .52 |  |  | 1.15 (0.92-1.42) | .22 |  | |  | | 0.52 (0.38-0.70) | <.001 |  |
|  | Age-adjusted |  | 0.97 (0.77-1.22) | .78 |  |  | 1.20 (0.96-1.49) | .10 |  | |  | | 0.85 (0.62-1.16) | .32 |  |
|  | Multivariable model^c^ |  | 0.92 (0.71-1.17) | .48 |  |  | 1.17 (0.94-1.45) | .16 |  | |  | | 0.91 (0.67-1.24) | .55 |  |
| Women  n_events_/n_total_ = 18/2796 | |  |  |  |  |  |  |  |  | |  | |  |  |  |
|  | Crude |  | 1.02 (0.66-1.59) | .92 |  |  | 1.77 (1.14-2.77) | .01 |  | |  | | 0.89 (0.49-1.62) | .70 |  |
|  | Age-adjusted |  | 1.01 (0.66-1.54) | .98 |  |  | 1.58 (1.03-2.43) | .04 |  | |  | | 0.98 (0.54-1.76) | .94 |  |
|  | Multivariable model^c^ |  | 1.17 (0.75-1.82) | .49 |  |  | 1.40 (0.87-2.27) | .17 |  | |  | | 0.90 (0.49-1.66) | .73 |  |
| Abbreviations: CI, confidence interval; FT_3_, free triiodothyronine; FT_4_, free thyroxine; HR, hazard ratio; TSH, thyrotropin.  Point estimates are expressed per SD increment in the predictor.  ^a^Evidence against the null hypothesis of no effect modification by sex on the association of the thyroid function parameter and the specific outcome concerned.  ^b^Adjusted for age, sex, current smoking, body-mass index, systolic blood pressure, use of antihypertensive drugs, total cholesterol/high-density lipoprotein ratio, ln triglycerides, use of lipid-lowering drugs, type 2 diabetes, high-sensitive C-reactive protein, history of cardiovascular disease, estimated glomerular filtration rate, and ln urinary albumin excretion.  ^c^Adjusted for all potential confounders except for sex. | | | | | | | | | | | | | | | |

| Supplementary Table 8: Total and age-stratified prospective associations of thyrotropin and thyroid function with all-cause and cardiovascular mortality after exclusion of 276 (4.5%) participants with incident malignancy since the previous screening | | | | | | | | | | | | | |
| --- | --- | --- | --- | --- | --- | --- | --- | --- | --- | --- | --- | --- | --- |
|  | | | | | | | | | | | | | |
|  |  |  | **TSH** | | |  | **FT_4_** | | |  | **FT_3_** | | |
| All-cause mortality | |  | **HR (95% CI)** | ***P* value** | ***P*_interaction_^a^** |  | **HR (95% CI)** | ***P* value** | ***P*_interaction_^a^** |  | **HR (95% CI)** | ***P* value** | ***P*_interaction_^a^** |
| Total population  n_events_/n_total_ = 345/5789 | |  |  |  |  |  |  |  |  |  |  |  |  |
|  | Crude |  | 0.93 (0.83-1.04) | .18 |  |  | 1.29 (1.16-1.43) | <.001 |  |  | 0.89 (0.78-1.02) | .09 |  |
|  | Age- and sex-adjusted |  | 0.99 (0.89-1.10) | .85 | .31 |  | 1.23 (1.11-1.37) | <.001 | .14 |  | 1.01 (0.88-1.16) | .88 | .88 |
|  | Multivariable model^b^ |  | 1.02 (0.91-1.14) | .73 | .56 |  | 1.17 (1.06-1.30) | .003 | .31 |  | 1.02 (0.89-1.17) | .81 | .98 |
| Low age (<70 years)^c^  n_events_/n_total_ = 172/5092 | |  |  |  |  |  |  |  |  |  |  |  |  |
|  | Crude |  | 0.85 (0.72-1.00) | .05 |  |  | 1.14 (0.98-1.33) | .08 |  |  | 1.04 (0.90-1.21) | .59 |  |
|  | Age- and sex-adjusted |  | 0.88 (0.75-1.03) | .12 |  |  | 1.13 (0.98-1.31) | .09 |  |  | 1.05 (0.88-1.26) | .59 |  |
|  | Multivariable model^b^ |  | 0.98 (0.83-1.15) | .78 |  |  | 1.10 (0.95-1.28) | .19 |  |  | 1.05 (0.87-1.26) | .62 |  |
| High age (≥70 years)^c^  n_events_/n_total_ = 173/697 | |  |  |  |  |  |  |  |  |  |  |  |  |
|  | Crude |  | 1.04 (0.90-1.20) | .59 |  |  | 1.35 (1.17-1.56) | <.001 |  |  | 1.04 (0.85-1.27) | .70 |  |
|  | Age- and sex-adjusted |  | 1.10 (0.96-1.27) | .18 |  |  | 1.35 (1.16-1.56) | <.001 |  |  | 0.99 (0.81-1.22) | .95 |  |
|  | Multivariable model^b^ |  | 1.07 (0.92-1.25) | .35 |  |  | 1.26 (1.08-1.46) | .003 |  |  | 1.01 (0.83-1.24) | .90 |  |
| Cardiovascular mortality | |  | **HR (95% CI)** | ***P* value** | ***P*_interaction_^a^** |  | **HR (95% CI)** | ***P* value** | ***P*_interaction_^a^** |  | **HR (95% CI)** | ***P* value** | ***P*_interaction_^a^** |
| Total population  n_events_/n_total_ = 96/5789 | |  |  |  |  |  |  |  |  |  |  |  |  |
|  | Crude |  | 0.89 (0.73-1.10) | .30 |  |  | 1.33 (1.09-1.62) | .004 |  |  | 0.74 (0.57-0.96) | .02 |  |
|  | Age- and sex-adjusted |  | 0.97 (0.79-1.19) | .78 | .07 |  | 1.26 (1.04-1.52) | .02 | .06 |  | 0.87 (0.66-1.14) | .31 | .17 |
|  | Multivariable model^b^ |  | 0.97 (0.78-1.20) | .75 | .02 |  | 1.17 (0.97-1.43) | .10 | .09 |  | 0.92 (0.70-1.22) | .57 | .31 |
| Low age (<72 years)^c^  n_events_/n_total_ = 48/5291 | |  |  |  |  |  |  |  |  |  |  |  |  |
|  | Crude |  | 1.14 (0.87-1.50) | .35 |  |  | 1.12 (0.84-1.48) | .46 |  |  | 1.01 (0.73-1.38) | .97 |  |
|  | Age- and sex-adjusted |  | 1.18 (0.91-1.54) | .21 |  |  | 1.09 (0.83-1.42) | .54 |  |  | 1.00 (0.69-1.45) | .99 |  |
|  | Multivariable model^b^ |  | 1.34 (1.02-1.77) | .04 |  |  | 1.00 (0.76-1.31) | .99 |  |  | 1.03 (0.71-1.50) | .88 |  |
| High age (≥72 years)^c^  n_events_/n_total_ = 48/498 | |  |  |  |  |  |  |  |  |  |  |  |  |
|  | Crude |  | 0.73 (0.53-1.01) | .05 |  |  | 1.45 (1.10-1.92) | .01 |  |  | 0.78 (0.51-1.18) | .23 |  |
|  | Age- and sex-adjusted |  | 0.75 (0.54-1.05) | .09 |  |  | 1.51 (1.12-2.03) | .007 |  |  | 0.73 (0.48-1.11) | .14 |  |
|  | Multivariable model^b^ |  | 0.68 (0.48-0.97) | .03 |  |  | 1.49 (1.09-2.04) | .01 |  |  | 0.81 (0.53-1.24) | .33 |  |
| Abbreviations: CI, confidence interval; FT_3_, free triiodothyronine; FT_4_, free thyroxine; HR, hazard ratio; TSH, thyrotropin.  Point estimates are expressed per SD increment in the predictor.  ^a^Evidence against the null hypothesis of no effect modification by age on the association of the thyroid function parameter and the specific outcome concerned.  ^b^Adjusted for age, sex, current smoking, body-mass index, systolic blood pressure, use of antihypertensive drugs, total cholesterol/high-density lipoprotein ratio, ln triglycerides, use of lipid-lowering drugs, type 2 diabetes, high-sensitive C-reactive protein, history of cardiovascular disease, estimated glomerular filtration rate, and ln urinary albumin excretion.  ^c^Cut-off values are established through dichotomisation of the cohort by the median age for half the events, to obtain an equal amount of events per stratum. | | | | | | | | | | | | | |

| Supplementary Table 9: Total and sex-stratified prospective associations of thyrotropin and thyroid function with all-cause and cardiovascular mortality after exclusion of 389 (6.4%) participants with pre-existing cardiovascular disease at baseline | | | | | | | | | | | | | | | |
| --- | --- | --- | --- | --- | --- | --- | --- | --- | --- | --- | --- | --- | --- | --- | --- |
|  | | | | | | | | | | | | | | | |
|  |  |  | **TSH** | | |  | **FT_4_** | | |  | | **FT_3_** | | | |
| All-cause mortality | |  | **HR (95% CI)** | ***P* value** | ***P*_interaction_^a^** |  | **HR (95% CI)** | ***P* value** | ***P*_interaction_^a^** | |  | | **HR (95% CI)** | ***P* value** | ***P*_interaction_^a^** |
| Total population  n_events_/n_total_ = 283/5665 | |  |  |  |  |  |  |  |  | |  | |  |  |  |
|  | Crude |  | 0.96 (0.85-1.08) | .48 |  |  | 1.24 (1.10-1.39) | <.001 |  | |  | | 0.87 (0.75-1.01) | .06 |  |
|  | Age- and sex-adjusted |  | 1.01 (0.90-1.14) | .85 | .82 |  | 1.22 (1.09-1.37) | .001 | .04 | |  | | 0.98 (0.86-1.11) | .72 | .001 |
|  | Multivariable model^b^ |  | 1.04 (0.92-1.17) | .53 | .77 |  | 1.20 (1.06-1.35) | .003 | .03 | |  | | 0.97 (0.85-1.10) | .63 | .001 |
| Men  n_events_/n_total_ = 202/2855 | |  |  |  |  |  |  |  |  | |  | |  |  |  |
|  | Crude |  | 0.99 (0.86-1.15) | .93 |  |  | 1.04 (0.91-1.20) | .55 |  | |  | | 0.56 (0.46-0.67) | <.001 |  |
|  | Age-adjusted |  | 1.02 (0.89-1.18) | .74 |  |  | 1.13 (0.99-1.30) | .07 |  | |  | | 0.86 (0.71-1.04) | .11 |  |
|  | Multivariable model^c^ |  | 1.03 (0.89-1.19) | .71 |  |  | 1.14 (0.99-1.31) | .08 |  | |  | | 0.86 (0.71-1.03) | .10 |  |
| Women  n_events_/n_total_ = 81/2810 | |  |  |  |  |  |  |  |  | |  | |  |  |  |
|  | Crude |  | 1.01 (0.82-1.25) | .91 |  |  | 1.62 (1.31-2.01) | <.001 |  | |  | | 1.12 (1.00-1.25) | .05 |  |
|  | Age-adjusted |  | 1.00 (0.81-1.22) | .98 |  |  | 1.51 (1.23-1.87) | <.001 |  | |  | | 1.19 (1.06-1.33) | .002 |  |
|  | Multivariable model^c^ |  | 1.05 (0.85-1.29) | .65 |  |  | 1.47 (1.18-1.82) | .001 |  | |  | | 1.19 (1.04-1.36) | .01 |  |
| Cardiovascular mortality | |  | **HR (95% CI)** | ***P* value** | ***P*_interaction_^a^** |  | **HR (95% CI)** | ***P* value** | ***P*_interaction_^a^** | |  | | **HR (95% CI)** | ***P* value** | ***P*_interaction_^a^** |
| Total population  n_events_/n_total_ = 66/5665 | |  |  |  |  |  |  |  |  | |  | |  |  |  |
|  | Crude |  | 0.96 (0.75-1.22) | .72 |  |  | 1.29 (1.02-1.64) | .04 |  | |  | | 0.61 (0.45-0.84) | .002 |  |
|  | Age- and sex-adjusted |  | 1.02 (0.80-1.30) | .85 | .96 |  | 1.27 (1.00-1.60) | .05 | .58 | |  | | 0.74 (0.53-1.03) | .08 | .07 |
|  | Multivariable model^b^ |  | 1.03 (0.80-1.32) | .84 | .92 |  | 1.31 (1.02-1.68) | .03 | .55 | |  | | 0.80 (0.58-1.10) | .17 | .21 |
| Men  n_events_/n_total_ = 49/2855 | |  |  |  |  |  |  |  |  | |  | |  |  |  |
|  | Crude |  | 0.99 (0.73-1.34) | .95 |  |  | 1.11 (0.84-1.46) | .48 |  | |  | | 0.38 (0.26-0.55) | <.001 |  |
|  | Age-adjusted |  | 1.03 (0.77-1.38) | .82 |  |  | 1.23 (0.93-1.62) | .16 |  | |  | | 0.65 (0.44-0.97) | .04 |  |
|  | Multivariable model^c^ |  | 0.95 (0.69-1.30) | .74 |  |  | 1.38 (1.04-1.82) | .03 |  | |  | | 0.72 (0.49-1.06) | .09 |  |
| Women  n_events_/n_total_ = 17/2810 | |  |  |  |  |  |  |  |  | |  | |  |  |  |
|  | Crude |  | 1.03 (0.66-1.62) | .89 |  |  | 1.64 (1.03-2.61) | .04 |  | |  | | 1.00 (0.60-1.68) | .99 |  |
|  | Age-adjusted |  | 1.01 (0.65-1.57) | .95 |  |  | 1.48 (0.95-2.32) | .09 |  | |  | | 1.09 (0.66-1.80) | .73 |  |
|  | Multivariable model^c^ |  | 1.22 (0.77-1.93) | .41 |  |  | 1.40 (0.87-2.26) | .17 |  | |  | | 1.06 (0.61-1.85) | .83 |  |
| Abbreviations: CI, confidence interval; FT_3_, free triiodothyronine; FT_4_, free thyroxine; HR, hazard ratio; TSH, thyrotropin.  Point estimates are expressed per SD increment in the predictor.  ^a^Evidence against the null hypothesis of no effect modification by sex on the association of the thyroid function parameter and the specific outcome concerned.  ^b^Adjusted for age, sex, current smoking, body-mass index, systolic blood pressure, use of antihypertensive drugs, total cholesterol/high-density lipoprotein ratio, ln triglycerides, use of lipid-lowering drugs, type 2 diabetes, high-sensitive C-reactive protein, estimated glomerular filtration rate, and ln urinary albumin excretion.  ^c^Adjusted for all potential confounders except for sex. | | | | | | | | | | | | | | | |

| Supplementary Table 10: Total and age-stratified prospective associations of thyrotropin and thyroid function with all-cause and cardiovascular mortality after exclusion of 389 (6.4%) participants with pre-existing cardiovascular disease at baseline | | | | | | | | | | | | | |
| --- | --- | --- | --- | --- | --- | --- | --- | --- | --- | --- | --- | --- | --- |
|  | | | | | | | | | | | | | |
|  |  |  | **TSH** | | |  | **FT_4_** | | |  | **FT_3_** | | |
| All-cause mortality | |  | **HR (95% CI)** | ***P* value** | ***P*_interaction_^a^** |  | **HR (95% CI)** | ***P* value** | ***P*_interaction_^a^** |  | **HR (95% CI)** | ***P* value** | ***P*_interaction_^a^** |
| Total population  n_events_/n_total_ = 283/5665 | |  |  |  |  |  |  |  |  |  |  |  |  |
|  | Crude |  | 0.96 (0.85-1.08) | .48 |  |  | 1.24 (1.10-1.39) | <.001 |  |  | 0.87 (0.75-1.01) | .06 |  |
|  | Age- and sex-adjusted |  | 1.01 (0.90-1.14) | .85 | .34 |  | 1.22 (1.09-1.37) | .001 | .47 |  | 0.98 (0.86-1.11) | .72 | .46 |
|  | Multivariable model^b^ |  | 1.04 (0.92-1.17) | .53 | .48 |  | 1.20 (1.06-1.35) | .003 | .56 |  | 0.97 (0.85-1.10) | .63 | .67 |
| Low age (<69 years)^c^  n_events_/n_total_ = 141/5026 | |  |  |  |  |  |  |  |  |  |  |  |  |
|  | Crude |  | 0.88 (0.74-1.05) | .15 |  |  | 1.16 (0.98-1.37) | .08 |  |  | 1.02 (0.82-1.19) | .75 |  |
|  | Age- and sex-adjusted |  | 0.90 (0.76-1.07) | .23 |  |  | 1.17 (0.99-1.37) | .06 |  |  | 1.01 (0.89-1.13) | .91 |  |
|  | Multivariable model^b^ |  | 0.97 (0.82-1.16) | .75 |  |  | 1.17 (0.99-1.38) | .14 |  |  | 0.99 (0.86-1.13) | .84 |  |
| High age (≥69 years)^c^  n_events_/n_total_ = 142/639 | |  |  |  |  |  |  |  |  |  |  |  |  |
|  | Crude |  | 1.04 (0.89-1.22) | .60 |  |  | 1.28 (1.09-1.50) | .003 |  |  | 1.01 (0.81-1.26) | .94 |  |
|  | Age- and sex-adjusted |  | 1.13 (0.96-1.33) | .13 |  |  | 1.30 (1.10-1.54) | .002 |  |  | 0.96 (0.77-1.21) | .74 |  |
|  | Multivariable model^b^ |  | 1.12 (0.95-1.32) | .19 |  |  | 1.28 (1.08-1.52) | .005 |  |  | 1.00 (0.80-1.24) | .99 |  |
| Cardiovascular mortality | |  | **HR (95% CI)** | ***P* value** | ***P*_interaction_^a^** |  | **HR (95% CI)** | ***P* value** | ***P*_interaction_^a^** |  | **HR (95% CI)** | ***P* value** | ***P*_interaction_^a^** |
| Total population  n_events_/n_total_ = 66/5665 | |  |  |  |  |  |  |  |  |  |  |  |  |
|  | Crude |  | 0.96 (0.75-1.22) | .72 |  |  | 1.29 (1.02-1.64) | .04 |  |  | 0.61 (0.45-0.84) | .002 |  |
|  | Age- and sex-adjusted |  | 1.02 (0.80-1.30) | .85 | .02 |  | 1.27 (1.00-1.60) | .05 | .04 |  | 0.74 (0.53-1.03) | .08 | .03 |
|  | Multivariable model^b^ |  | 1.03 (0.80-1.32) | .84 | .005 |  | 1.31 (1.02-1.68) | .03 | .04 |  | 0.80 (0.58-1.10) | .17 | .20 |
| Low age (<73 years)^c^  n_events_/n_total_ = 33/5330 | |  |  |  |  |  |  |  |  |  |  |  |  |
|  | Crude |  | 1.27 (0.92-1.74) | .15 |  |  | 1.11 (0.78-1.56) | .57 |  |  | 0.68 (0.44-1.05) | .08 |  |
|  | Age- and sex-adjusted |  | 1.30 (0.95-1.78) | .10 |  |  | 1.09 (0.78-1.51) | .61 |  |  | 0.70 (0.44-1.11) | .13 |  |
|  | Multivariable model^b^ |  | 1.28 (0.93-1.76) | .13 |  |  | 1.22 (0.87-1.72) | .24 |  |  | 0.74 (0.48-1.13) | .17 |  |
| High age (≥73 years)^c^  n_events_/n_total_ = 33/335 | |  |  |  |  |  |  |  |  |  |  |  |  |
|  | Crude |  | 0.74 (0.50-1.09) | .12 |  |  | 1.48 (1.05-2.08) | .03 |  |  | 0.87 (0.54-1.41) | .57 |  |
|  | Age- and sex-adjusted |  | 0.74 (0.50-1.10) | .14 |  |  | 1.60 (1.12-2.30) | .01 |  |  | 0.82 (0.51-1.32) | .41 |  |
|  | Multivariable model^b^ |  | 0.68 (0.44-1.05) | .08 |  |  | 1.73 (1.16-2.57) | .007 |  |  | 0.92 (0.58-1.46) | .91 |  |
| Abbreviations: CI, confidence interval; FT_3_, free triiodothyronine; FT_4_, free thyroxine; HR, hazard ratio; TSH, thyrotropin.  Point estimates are expressed per SD increment in the predictor.  ^a^Evidence against the null hypothesis of no effect modification by age on the association of the thyroid function parameter and the specific outcome concerned.  ^b^Adjusted for age, sex, current smoking, body-mass index, systolic blood pressure, use of antihypertensive drugs, total cholesterol/high-density lipoprotein ratio, ln triglycerides, use of lipid-lowering drugs, type 2 diabetes, high-sensitive C-reactive protein, estimated glomerular filtration rate, and ln urinary albumin excretion.  ^c^Cut-off values are established through dichotomisation of the cohort by the median age for half the events, to obtain an equal amount of events per stratum. | | | | | | | | | | | | | |

| Supplementary Table 11: Total and sex-stratified prospective associations of thyrotropin and thyroid function with all-cause and cardiovascular mortality after exclusion of 51 (13.4%) deaths from all-causes and 20 (19.4%) from cardiovascular disease during the first two years of follow-up | | | | | | | | | | | | | | | |
| --- | --- | --- | --- | --- | --- | --- | --- | --- | --- | --- | --- | --- | --- | --- | --- |
|  |  |  | **TSH** | | |  | **FT_4_** | | |  | | **FT_3_** | | | |
| All-cause mortality | |  | **HR (95% CI)** | ***P* value** | ***P*_interaction_^a^** |  | **HR (95% CI)** | ***P* value** | ***P*_interaction_^a^** | |  | | **HR (95% CI)** | ***P* value** | ***P*_interaction_^a^** |
| Total population  n_events_/n_total_ = 329/6003 | |  |  |  |  |  |  |  |  | |  | |  |  |  |
|  | Crude |  | 0.95 (0.85-1.06) | .33 |  |  | 1.26 (1.13-1.40) | <.001 |  | |  | | 0.87 (0.76-1.00) | .04 |  |
|  | Age- and sex-adjusted |  | 1.01 (0.90-1.12) | .89 | .59 |  | 1.22 (1.10-1.35) | <.001 | .15 | |  | | 0.98 (0.87-1.10) | .71 | .001 |
|  | Multivariable model^b^ |  | 1.05 (0.94-1.17) | .43 | .64 |  | 1.17 (1.05-1.30) | .004 | .08 | |  | | 0.97 (0.86-1.10) | .67 | .002 |
| Men  n_events_/n_total_ = 247/3108 | |  |  |  |  |  |  |  |  | |  | |  |  |  |
|  | Crude |  | 0.96 (0.83-1.09) | .50 |  |  | 1.10 (0.97-1.25) | .12 |  | |  | | 0.57 (0.48-0.68) | <.001 |  |
|  | Age-adjusted |  | 0.99 (0.87-1.13) | .90 |  |  | 1.17 (1.04-1.32) | .01 |  | |  | | 0.88 (0.74-1.05) | .15 |  |
|  | Multivariable model^c^ |  | 1.00 (0.88-1.14) | >.99 |  |  | 1.15 (1.02-1.30) | .03 |  | |  | | 0.88 (0.74-1.04) | .13 |  |
| Women  n_events_/n_total_ = 82/2895 | |  |  |  |  |  |  |  |  | |  | |  |  |  |
|  | Crude |  | 1.08 (0.88-1.32) | .45 |  |  | 1.53 (1.23-1.91) | <.001 |  | |  | | 1.12 (0.99-1.25) | .06 |  |
|  | Age-adjusted |  | 1.06 (0.87-1.29) | .56 |  |  | 1.44 (1.16-1.78) | <.001 |  | |  | | 1.19 (1.06-1.33) | .004 |  |
|  | Multivariable model^c^ |  | 1.16 (0.95-1.43) | .14 |  |  | 1.35 (1.08-1.70) | .009 |  | |  | | 1.18 (1.03-1.36) | .02 |  |
| Cardiovascular mortality | |  | **HR (95% CI)** | ***P* value** | ***P*_interaction_^a^** |  | **HR (95% CI)** | ***P* value** | ***P*_interaction_^a^** | |  | | **HR (95% CI)** | ***P* value** | ***P*_interaction_^a^** |
| Total population  n_events_/n_total_ = 83/6034 | |  |  |  |  |  |  |  |  | |  | |  |  |  |
|  | Crude |  | 0.94 (0.76-1.18) | .60 |  |  | 1.35 (1.10-1.67) | .005 |  | |  | | 0.75 (0.57-0.99) | .04 |  |
|  | Age- and sex-adjusted |  | 1.03 (0.83-1.28) | .80 | .18 |  | 1.29 (1.05-1.59) | .02 | .64 | |  | | 0.87 (0.65-1.17) | .37 | .44 |
|  | Multivariable model^b^ |  | 1.05 (0.84-1.32) | .64 | .16 |  | 1.22 (0.99-1.51) | .06 | .84 | |  | | 0.92 (0.68-1.23) | .56 | .61 |
| Men  n_events_/n_total_ = 69/3135 | |  |  |  |  |  |  |  |  | |  | |  |  |  |
|  | Crude |  | 0.91 (0.70-1.18) | .47 |  |  | 1.24 (0.99-1.56) | .06 |  | |  | | 0.51 (0.37-0.70) | <.001 |  |
|  | Age-adjusted |  | 0.96 (0.75-1.22) | .72 |  |  | 1.33 (1.06-1.67) | .02 |  | |  | | 0.85 (0.61-1.19) | .35 |  |
|  | Multivariable model^c^ |  | 0.92 (0.71-1.20) | .55 |  |  | 1.29 (1.02-1.63) | .03 |  | |  | | 0.88 (0.63-1.23) | .44 |  |
| Women  n_events_/n_total_ = 14/2899 | |  |  |  |  |  |  |  |  | |  | |  |  |  |
|  | Crude |  | 1.42 (0.89-2.24) | .14 |  |  | 1.31 (0.76-2.24) | .33 |  | |  | | 0.97 (0.52-1.81) | .92 |  |
|  | Age-adjusted |  | 1.38 (0.87-2.16) | .17 |  |  | 1.21 (0.72-2.04) | .47 |  | |  | | 1.05 (0.57-1.92) | .88 |  |
|  | Multivariable model^c^ |  | 1.77 (1.05-2.99) | .03 |  |  | 1.13 (0.64-1.99) | .68 |  | |  | | 0.99 (0.52-1.86) | .97 |  |
| Abbreviations: CI, confidence interval; FT_3_, free triiodothyronine; FT_4_, free thyroxine; HR, hazard ratio; TSH, thyrotropin.  Point estimates are expressed per SD increment in the predictor.  ^a^Evidence against the null hypothesis of no effect modification by sex on the association of the thyroid function parameter and the specific outcome concerned.  ^b^Adjusted for age, sex, current smoking, body-mass index, systolic blood pressure, use of antihypertensive drugs, total cholesterol/high-density lipoprotein ratio, ln triglycerides, use of lipid-lowering drugs, type 2 diabetes, high-sensitive C-reactive protein, estimated glomerular filtration rate, and ln urinary albumin excretion.  ^c^Adjusted for all potential confounders except for sex. | | | | | | | | | | | | | | | |

| Supplementary Table 12: Total and age-stratified prospective associations of thyrotropin and thyroid function with all-cause and cardiovascular mortality after exclusion of 51 (13.4%) deaths from all-causes and 20 (19.4%) from cardiovascular disease during the first two years of follow-up | | | | | | | | | | | | | |
| --- | --- | --- | --- | --- | --- | --- | --- | --- | --- | --- | --- | --- | --- |
|  | | | | | | | | | | | | | |
|  |  |  | **TSH** | | |  | **FT_4_** | | |  | **FT_3_** | | |
| All-cause mortality | |  | **HR (95% CI)** | ***P* value** | ***P*_interaction_^a^** |  | **HR (95% CI)** | ***P* value** | ***P*_interaction_^a^** |  | **HR (95% CI)** | ***P* value** | ***P*_interaction_^a^** |
| Total population  n_events_/n_total_ = 329/6003 | |  |  |  |  |  |  |  |  |  |  |  |  |
|  | Crude |  | 0.95 (0.85-1.06) | .33 |  |  | 1.26 (1.13-1.40) | <.001 |  |  | 0.87 (0.76-1.00) | .04 |  |
|  | Age- and sex-adjusted |  | 1.01 (0.90-1.12) | .89 | .22 |  | 1.22 (1.10-1.35) | <.001 | .40 |  | 0.98 (0.87-1.10) | .71 | .54 |
|  | Multivariable model^b^ |  | 1.05 (0.94-1.17) | .43 | .39 |  | 1.17 (1.05-1.30) | .004 | .71 |  | 0.97 (0.86-1.10) | .67 | .53 |
| Low age (<69 years)^c^  n_events_/n_total_ = 164/5256 | |  |  |  |  |  |  |  |  |  |  |  |  |
|  | Crude |  | 0.85 (0.72-1.01) | .06 |  |  | 1.18 (1.01-1.37) | .04 |  |  | 1.02 (0.89-1.16) | .79 |  |
|  | Age- and sex-adjusted |  | 0.89 (0.75-1.04) | .14 |  |  | 1.17 (1.01-1.36) | .04 |  |  | 1.00 (0.90-1.12) | .97 |  |
|  | Multivariable model^b^ |  | 1.00 (0.85-1.17) | .98 |  |  | 1.14 (0.98-1.33) | .08 |  |  | 1.00 (0.88-1.13) | .99 |  |
| High age (≥69 years)^c^  n_events_/n_total_ = 165/747 | |  |  |  |  |  |  |  |  |  |  |  |  |
|  | Crude |  | 1.05 (0.91-1.21) | .49 |  |  | 1.28 (1.10-1.49) | .001 |  |  | 0.99 (0.80-1.22) | .94 |  |
|  | Age- and sex-adjusted |  | 1.13 (0.98-1.31) | .09 |  |  | 1.29 (1.10-1.50) | .001 |  |  | 0.94 (0.76-1.17) | .59 |  |
|  | Multivariable model^b^ |  | 1.10 (0.95-1.28) | .20 |  |  | 1.23 (1.05-1.44) | .009 |  |  | 0.97 (0.78-1.20) | .75 |  |
| Cardiovascular mortality | |  | **HR (95% CI)** | ***P* value** | ***P*_interaction_^a^** |  | **HR (95% CI)** | ***P* value** | ***P*_interaction_^a^** |  | **HR (95% CI)** | ***P* value** | ***P*_interaction_^a^** |
| Total population  n_events_/n_total_ = 83/6034 | |  |  |  |  |  |  |  |  |  |  |  |  |
|  | Crude |  | 0.94 (0.76-1.18) | .60 |  |  | 1.35 (1.10-1.67) | .005 |  |  | 0.75 (0.57-0.99) | .04 |  |
|  | Age- and sex-adjusted |  | 1.03 (0.83-1.28) | .80 | .14 |  | 1.29 (1.05-1.59) | .02 | .07 |  | 0.87 (0.65-1.17) | .37 | .56 |
|  | Multivariable model^b^ |  | 1.05 (0.84-1.32) | .64 | .06 |  | 1.22 (0.99-1.51) | .06 | .12 |  | 0.92 (0.68-1.23) | .56 | .58 |
| Low age (<71 years)^c^  n_events_/n_total_ = 41/5485 | |  |  |  |  |  |  |  |  |  |  |  |  |
|  | Crude |  | 1.06 (0.78-1.43) | .72 |  |  | 1.13 (0.83-1.54) | .44 |  |  | 0.99 (0.73-1.35) | .96 |  |
|  | Age- and sex-adjusted |  | 1.12 (0.83-1.50) | .46 |  |  | 1.12 (0.83-1.50) | .46 |  |  | 0.99 (0.76-1.28) | .93 |  |
|  | Multivariable model^b^ |  | 1.31 (0.97-1.78) | .08 |  |  | 1.01 (0.75-1.35) | .97 |  |  | 1.01 (0.79-1.29) | .92 |  |
| High age (≥71 years)^c^  n_events_/n_total_ = 42/549 | |  |  |  |  |  |  |  |  |  |  |  |  |
|  | Crude |  | 0.89 (0.65-1.21) | .45 |  |  | 1.45 (1.08-1.94) | .12 |  |  | 0.76 (0.49-1.18) | .23 |  |
|  | Age- and sex-adjusted |  | 0.94 (0.69-1.29) | .71 |  |  | 1.55 (1.13-2.13) | .06 |  |  | 0.72 (0.46-1.14) | .16 |  |
|  | Multivariable model^b^ |  | 0.88 (0.63-1.23) | .45 |  |  | 1.52 (1.10-2.12) | .12 |  |  | 0.78 (0.49-1.23) | .28 |  |
| Abbreviations: CI, confidence interval; FT_3_, free triiodothyronine; FT_4_, free thyroxine; HR, hazard ratio; TSH, thyrotropin.  Point estimates are expressed per SD increment in the predictor.  ^a^Evidence against the null hypothesis of no effect modification by age on the association of the thyroid function parameter and the specific outcome concerned.  ^b^Adjusted for age, sex, current smoking, body-mass index, systolic blood pressure, use of antihypertensive drugs, total cholesterol/high-density lipoprotein ratio, ln triglycerides, use of lipid-lowering drugs, type 2 diabetes, high-sensitive C-reactive protein, estimated glomerular filtration rate, and ln urinary albumin excretion.  ^c^Cut-off values are established through dichotomisation of the cohort by the median age for half the events, to obtain an equal amount of events per stratum. | | | | | | | | | | | | | |

| Supplementary Table 13: Total and age-stratified prospective associations of thyrotropin and thyroid function with all-cause and cardiovascular mortality after stratification by the age of 65 years | | | | | | | | | | | | | |
| --- | --- | --- | --- | --- | --- | --- | --- | --- | --- | --- | --- | --- | --- |
|  | | | | | | | | | | | | | |
|  |  |  | **TSH** | | |  | **FT_4_** | | |  | **FT_3_** | | |
| All-cause mortality | |  | **HR (95% CI)** | ***P* value** | ***P*_interaction_^a^** |  | **HR (95% CI)** | ***P* value** | ***P*_interaction_^a^** |  | **HR (95% CI)** | ***P* value** | ***P*_interaction_^a^** |
| Total population  n_events_/n_total_ = 380/6054 | |  |  |  |  |  |  |  |  |  |  |  |  |
|  | Crude |  | 0.93 (0.84-1.03) | .18 |  |  | 1.29 (1.17-1.42) | <.001 |  |  | 0.88 (0.78-1.00) | .05 |  |
|  | Age- and sex-adjusted |  | 0.99 (0.90-1.10) | .89 | .31 |  | 1.24 (1.13-1.37) | <.001 | .21 |  | 0.99 (0.89-1.09) | .81 | .81 |
|  | Multivariable model^b^ |  | 1.02 (0.92-1.14) | .65 | .55 |  | 1.18 (1.07-1.30) | .001 | .46 |  | 0.99 (0.89-1.10) | .81 | .85 |
| Low age (<65 years)  n_events_/n_total_ = 122/4810 | |  |  |  |  |  |  |  |  |  |  |  |  |
|  | Crude |  | 0.89 (0.74-1.07) | .22 |  |  | 1.12 (0.93-1.34) | .24 |  |  | 1.06 (0.90-1.25) | .49 |  |
|  | Age- and sex-adjusted |  | 0.90 (0.74-1.08) | .25 |  |  | 1.14 (0.95-1.36) | .15 |  |  | 1.06 (0.86-1.30) | .59 |  |
|  | Multivariable model^b^ |  | 1.03 (0.86-1.25) | .73 |  |  | 1.13 (0.94-1.36) | .20 |  |  | 1.04 (0.84-1.28) | .74 |  |
| High age (≥65 years)  n_events_/n_total_ = 258/1244 | |  |  |  |  |  |  |  |  |  |  |  |  |
|  | Crude |  | 0.99 (0.88-1.11) | .84 |  |  | 1.30 (1.16-1.45) | <.001 |  |  | 0.99 (0.89-1.10) | .79 |  |
|  | Age- and sex-adjusted |  | 1.04 (0.93-1.18) | .48 |  |  | 1.30 (1.15-1.46) | <.001 |  |  | 0.97 (0.86-1.11) | .69 |  |
|  | Multivariable model^b^ |  | 1.01 (0.90-1.15) | .82 |  |  | 1.24 (1.10-1.40) | <.001 |  |  | 0.99 (0.88-1.11) | .83 |  |
| Cardiovascular mortality | |  | **HR (95% CI)** | ***P* value** | ***P*_interaction_^a^** |  | **HR (95% CI)** | ***P* value** | ***P*_interaction_^a^** |  | **HR (95% CI)** | ***P* value** | ***P*_interaction_^a^** |
| Total population  n_events_/n_total_ = 103/6054 | |  |  |  |  |  |  |  |  |  |  |  |  |
|  | Crude |  | 0.89 (0.73-1.09) | .26 |  |  | 1.40 (1.16-1.69) | .001 |  |  | 0.73 (0.57-0.93) | .01 |  |
|  | Age- and sex-adjusted |  | 0.97 (0.79-1.18) | .75 | .09 |  | 1.34 (1.11-1.61) | .002 | .03 |  | 0.86 (0.66-1.12) | .27 | .16 |
|  | Multivariable model^b^ |  | 0.98 (0.80-1.20) | .86 | .04 |  | 1.23 (1.02-1.49) | .03 | .06 |  | 0.91 (0.70-1.18) | .48 | .28 |
| Low age (<65 years)  n_events_/n_total_ = 23/4810 | |  |  |  |  |  |  |  |  |  |  |  |  |
|  | Crude |  | 1.05 (0.70-1.58) | .80 |  |  | 0.88 (0.57-1.36) | .57 |  |  | 1.08 (0.78-1.51) | .64 |  |
|  | Age- and sex-adjusted |  | 1.07 (0.72-1.60) | .74 |  |  | 0.90 (0.60-1.38) | .65 |  |  | 1.09 (0.68-1.76) | .72 |  |
|  | Multivariable model^b^ |  | 1.38 (0.91-2.11) | .13 |  |  | 0.90 (0.57-1.42) | .65 |  |  | 1.11 (0.70-1.77) | .66 |  |
| High age (≥65 years)  n_events_/n_total_ = 80/1244 | |  |  |  |  |  |  |  |  |  |  |  |  |
|  | Crude |  | 0.89 (0.71-1.11) | .31 |  |  | 1.47 (1.20-1.79) | <.001 |  |  | 0.81 (0.60-1.09) | .17 |  |
|  | Age- and sex-adjusted |  | 0.94 (0.75-1.18) | .59 |  |  | 1.49 (1.21-1.84) | <.001 |  |  | 0.79 (0.58-1.09) | .15 |  |
|  | Multivariable model^b^ |  | 0.90 (0.71-1.14) | .39 |  |  | 1.38 (1.12-1.72) | .003 |  |  | 0.85 (0.62-1.16) | .31 |  |
| Abbreviations: CI, confidence interval; FT_3_, free triiodothyronine; FT_4_, free thyroxine; HR, hazard ratio; TSH, thyrotropin.  Point estimates are expressed per SD increment in the predictor.  ^a^Evidence against the null hypothesis of no effect modification by age on the association of the thyroid function parameter and the specific outcome concerned.  ^b^Adjusted for age, sex, current smoking, body-mass index, systolic blood pressure, use of antihypertensive drugs, total cholesterol/high-density lipoprotein ratio, ln triglycerides, use of lipid-lowering drugs, type 2 diabetes, high-sensitive C-reactive protein, estimated glomerular filtration rate, and ln urinary albumin excretion. | | | | | | | | | | | | | |
